# A lightweight hybrid deep learning approach for fashion mnist classification with explainable attention visualization

**DOI:** 10.1371/journal.pone.0351671

**Published:** 2026-06-23

**Authors:** Hafeez Ahmad, Tahira Anwar Lashari, Saima Anwar Lashari, Ijaz Khan, Farzana Jabeen

**Affiliations:** 1 School of Electrical Engineering and Computer Science, National University of Sciences & Technology (NUST), Islamabad, Pakistan; 2 College of Computing and Informatics, Saudi Electronic University, Riyadh, Saudi Arabia; 3 College of Aeronautical Engineering, Military College of Engineering NUST, Risalpur, Pakistan; Najran University College of Computer Science and Information Systems, SAUDI ARABIA

## Abstract

The classification of fashion images is an essential task in the e-commerce sector, where accurate categorization improves user experience and refines product discovery. Convolutional Neural Networks (CNNs) and Transformers have demonstrated strong performance in image classification tasks due to their ability to learn complex visual features. However, deep variants of these architectures, such as VGG-19, ResNet-50, Vision Transformer (ViT), and Swin Transformer, contain tens of millions of parameters, requiring high memory and powerful GPUs for training, which makes them less suitable for low-resource and edge device environments. To address these limitations, this research proposes a lightweight hybrid architecture, TinyCNN with Linear Self-Attention (LSA), optimized for resource-constrained settings. The proposed model contains fewer than half a million parameters and is trainable on a CPU, achieving a classification accuracy of 91.47% on the Fashion-MNIST dataset. In addition, multiple Explainable Artificial Intelligence (XAI) techniques are implemented, including Self-Attention visualization, Multi-Head Attention, Attention Flow, Attention Rollout, Fixed query position attention maps, Integrated Gradients, LIME, and SHAP, to provide visual interpretability of the model’s predictions and enhance transparency in its decision-making process.

## Introduction

Machine learning systems are now being more widely integrated in various applications, ranging from e-commerce platforms, to the textile industry. Machine learning is the key to developing AI models with clothes and other instances where machines learn to use such a model in order to identify critical properties like design, color, and shape of clothes. It enables users to understand the products better, reach a large customer base in different regions, ultimately resulting in more sales. Image classification is one of the fundamental tasks in Computer Vision and serves as an integral part of these systems. Although humans can easily identify whether these images depict pants, hats, or shoes, it is particularly difficult for machines to accurately perceive and classify clothing images. This challenge is exacerbated when the visual distinctions between clothing types are nuanced, creating a fine-grained classification problem that is especially complex.

Traditional machine learning algorithms often struggle to effectively handle large image datasets. Deep learning algorithms are suitable for large and complex datasets [1]. Deep learning has been beneficial in recognizing fashion images. This technology improves user satisfaction by providing personalized clothing recommendations, leading to higher revenue generation for businesses. The computational cost is a major hindrance in training deep learning models on image datasets [2]. This challenge highlights the growing need to build low-compute-cost algorithms for fashion image classification.

The grouping of images into pre-established categories is called image classification. For this, particular features need to be extracted from images that carry information, such as color, and shape. The classification can be accomplished using machine learning classifiers such as Decision Trees [3], K-Nearest Neighbours [4], Naive Bayes [5], Random Forest [6], Multilayer Perceptron [7], and Support Vector Machines [8]. Convolutional Neural Networks(CNN) are widely used to learn these features [9]. A CNN is a type of multi-layer neural network inspired by the mechanism of the optical system of living creatures. In the image classification task, CNN learns the output features from data and then performs the classification based on the predefined categories. CNNs often outperform traditional machine learning algorithms in image classification tasks, though they are also prone to overfitting issues [10].

## Related work

Fashion MNIST [11] is a benchmark dataset widely used by researchers for the clothing image classification task. Combined dynamic learning rate strategies were used with standard CNNs on the Fashion-MNIST dataset to mitigate overfitting and reduce model training time [12]. In order to prevent overfitting and enhance training effectiveness, dropout regularization [13] was used, which probabilistically deters connections between neurons to reduce dependence on specific input features. Exponential decay learning rate, batch size adjustment, and convolutional depth were used, leading to 93% peak classification accuracy.

Multiple convolutional neural network (MCNN-14) [14] used CNNs for the feature extraction through hierarchies, which is beneficial in visual domains like fashion, where the hierarchy of abstraction is important. This indicated that standard CNNs find it hard to cope with background noise and variations within the same class. The MCNN-14 framework adopted 14 convolutional layers in the form of parallel subnets to allow simultaneous extraction of lower- and higher-order image-level features. The model achieved an accuracy of 93.08%. The ensemble technique utilized produced increased accuracy and scalability, outperforming the constraints of single monolithic deep networks. This showed a precision-oriented CNN ensemble model focused on the task of fashion image classification.

CrossViTL2 [15], a vision transformer aimed at addressing common problems of overfitting in categorizing fashion images. This approach converts an image into patches and obtains the global context using self-attention. Using L2 regularization showed generalization by controlling excessively large weight parameters. K-fold cross-validation was used to mitigate class imbalance biases in classification and improve assessment reliability. The study revealed that the implementation of the L2 regularization improved the model’s classification accuracy since it increased the metric from 91.30% to 93.47%. These results support the study by Nguyen et al. [16] that L2 regularization improves transformer training stability and task robustness in performance.

Two architectures, CNN-C1 and CNN-C2 [17], were designed to study the effect of regularization methods on the performance of a model. The CNN-C1 model showed training loss decreasing from 0.8778 to 0.1332 and accuracy increasing from 69.19% to 95.22%, while validation loss dropped from 1.4893 to 0.3387 and accuracy rose from 66.98% to 88.95%, indicating overfitting. On the other hand, CNN-C2 used batch normalization and dropout layers and achieved a validation accuracy of 98.01%. As suggested in the study [18], batch normalization normalizes outputs of internal layers and improves convergence as well as makes the learning process more stable. The synergy of these approaches serves as the foundation of sound regularization in CNNs and plays a key role in the performance of CNN-C2.

Four CNN architectures, RMCNN1, RMCNN2, RMCNN3, and RMCNN4 [19], each of them implemented with dropout regularization. Intermittently during the training phase, dropout switches off neurons within the network, thereby reducing the correlation between features and improving generalization. The highest classification accuracy was 95.81%. The observed performance improvement was a result of the collaborative effect of dropout regularization and expanded convolutional layers, as both added more feature extraction possibilities and reduced the probability of overfitting.

A custom dataset of 5000 images [20] was divided into two main categories and 4 sub-categories. “Male” and “Female” were the main categories. “Ethnic,” “Casual,” “Formal,” and “Sportswear” were the sub-categories. The study proposed a hybrid model of CNNs and SVMs. CNNs were used to extract features like shapes, edges, and textures. These features were passed to the SVM. The activation functions of CNN helped to get a nonlinear representation of the input data from each layer. SVM used the features provided by the CNN component output and used them to make predictions of the clothing categories. This way, this hybrid model benefited from both the strengths of CNNs and SVMs.

The self-attentional Transformer [21] was solely based on the attention mechanisms and completely dispensed with convolutions and recurrence. Self-attention is a mechanism of attention linked with the different areas of a sequence with the aim of calculating the representation of the whole sequence. The architecture contains encoders and decoders. Transformers have the ability to process each and every part of the input sequence at the same time. In the self-attention mechanism, the model calculates a representation of a sequence by relating every word to every other word in the same sequence. Hence, the distance between any two positions is reduced to a constant number, irrespective of the distance between them which makes it easy to model the long-range dependencies. The Long Short-term memory and the Self-attention architecture were combined by Cheng et al. to represent sequence-level networks, which are good at managing structured input [22]. The attention mechanism was also used for semantic role labeling [23]. The attention mechanism has been widely used in the natural language processing tasks as well.

The classification of media painting images using an enhanced ResNet50 architecture addressed the challenge of accurately identifying artistic styles, often characterized by nuanced visual features that traditional classifiers struggle to capture [24]. To enhance focus on stylistic regions, a triplet attention mechanism was implemented that combined spatial, channel-wise, and branch-level attention. This allowed the network to adaptively concentrate on the most informative parts of an image. The model achieved 80.6% accuracy, and 11.7% improvement over standard ResNet50. The study demonstrated that integrating architectural enhancements with attention mechanisms produces more interpretable and accurate classification models.

For comparing the applicability of lightweight mobile-oriented convolutional architectures to Fashion-MNIST, Di compared four models under a unified experimental setting: Fully Connected Neural Network, CNN, MobileNetV1 and MobileNetV2 [25]. In particular, all experiments are conducted on the widely-used Fashion-MNIST dataset. The authors used cross-entropy loss, Adam optimizer, and batch size of 128 with 20 iterations for training all networks in order to ensure consistency across models. The paper also states that the models are all trained on GPU computation, and that kind of experimental platform is made using a NVIDIA GTX970 GPU + Intel i7-6700K. The original MobileNet architecture expects larger RGB image inputs; hence, the authors upscaled the Fashion-MNIST images from 28 × 28–56 × 56 and duplicated the grayscale channel three times before passing it through MobileNetV1 and MobileNetV2; this same resized input setting was used on all other models as well in order to maintain comparability across architectures under evaluation. In terms of the reported results, MobileNetV1 achieved a test accuracy of 91.12%, and MobileNetV2 achieved a test accuracy of 92.91%. MobileNetV1 employed 4.2M parameters, and MobileNetV2 used 3.4M parameters in terms of model size. Even so, both models still in the multi-million-parameter range suggests that the reported accuracy improvements are still realized with relatively large parameter budgets. This is particularly important in studies where the tightness of parameters, given the design choices, is treated as a fundamental principle rather than an object to be optimized as an afterthought. The MobileNetV1 and MobileNetV2 had runtimes of 83s and 98s, respectively, for the given hardware configuration. There is a nontrivial computational cost for each of the MobileNet-based models listed. Since these results were achieved with GPU acceleration in one specific hyper-parameter configuration on an NVIDIA GTX970, it may be concluded that while increased Fashion-MNIST classification power is found for MobileNet-family architectures this comes at a cost of not only multi-million parameter models but also higher computational burden. At the same time, its results show that the reported accuracy is achieved at 4.2M and 3.4M parameters for MobileNetV1 and MobileNetV2 respectively, leaving it an open question whether a competitive performance can be approached with considerably smaller parameter count.

Kumar proposed an adaptive fusion-based image classification framework that fuses features representations derived from VGG16, ResNet50 and MobileNetV2 and tested it on the Fashion-MNIST dataset [26]. The reported pipeline applied channel replication to the original 28×28 grayscale images to create three-channel inputs, resized them to 48×48 pixels and augmented the input via rotation before running feature extraction followed by concatenation and classification. It is trained with Adam and categorical cross-entropy, 10 epochs, batch size of 128. The study reported a training accuracy of 97.50% and validation accuracy of 89.27% while also observing fluctuations in validation loss that reflect overfitting.

Recent work has emphasized the need for designing deep learning based architectures that are robust in the face of distributional drift and changing characteristics. In the context of PM2.5 forecasting, Hossen et al. proposed a drift-aware deep learning framework that takes an explicit perspective on analyzing yearly data drift using statistical measures such as Jensen-Shannon divergence, maximum mean discrepancy, and Pearson correlation, which they exploit in combination with a wrapped loss function along with CNN-based neural architectures to enhance robustness under nonstationary environments [27]. The authors have built upon this approach with a follow-up study introducing ODE-based continuous-time neural models, including a transformer-based ODE model and a closed-form continuous-time framework that are able to better capture temporal dependency, leading to significant improvements in PM2.5 prediction accuracy improvement over LSTM-based baselines whilst further stressing the interpretability advantages of ODE-fueled system dynamics [28]. While these studies primarily center around environmental time-series prediction instead of image classification, they are relevant as they demonstrate how drift-aware learning, hybrid neural design, and more interpretable modeling strategies can aid in greater robustness for deep learning systems.

## Methodology

### Dataset description

This study used the Fashion MNIST dataset [11], a publicly available dataset on GitHub (https://github.com/zalandoresearch/fashion-mnist), and a well-known benchmark for the fashion image classification task. The Fashion MNIST dataset is publicly available. This was an appropriate dataset for our experimentation because it provides enough complexity for evaluating an attention-based model. Fashion MNIST dataset contains a total of 70,000 images across 10 clothing categories: T-shirt/top, Trouser, Pullover, Dress, Coat, Sandal, Shirt, Sneaker, Bag, and Ankle Boot. By default, the dataset comes with train and test partitions. The train set contains 60,000 images, and the test set contains 10,000 images. The images are greyscale with 28x28 pixels in dimensions. To enable model generalization capability, the original training set of the Fashion MNIST dataset was further divided into two non-overlapping subsets:

- **Training Subset:** 48,000 samples (80% of the original training set)
- **Validation Subset:** 12,000 samples (20% of the original training set)

### Proposed model architecture: TinyCNN with linear self-attention

Convolutional Neural Networks (CNNs) have been widely used for image classification tasks and have shown good performance on small to medium-sized datasets. However, CNNs face challenges in capturing long-range spatial dependencies, as their receptive field expands gradually with increasing depth. Transformers uses a self-attention mechanism to model global context, but this comes with a computational cost that increases quadratically with the number of tokens. Full self-attention is unneeded and computationally wasteful for small datasets such as Fashion MNIST (28 x 28 Grayscale). To achieve a balance between accuracy and computational cost, this study proposed a hybrid architecture called TinyCNN-LSA, which integrated the local feature extraction strengths of CNNs with the global context modeling abilities of Linear Self-Attention (LSA). The aim was to enable the model to reason over both local and global patterns efficiently.

### Architecture overview

**Fig 1:**
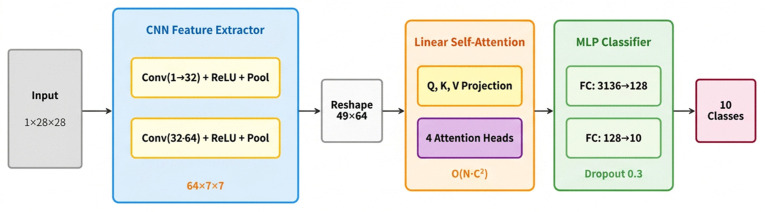
TinyCNN-LSA Architecture. The input image passes through a CNN Feature Extractor, Linear Self-Attention (LSA) module with 4 heads, and an MLP classifier with Dropout 0.3, yielding predictions over 10 classes.

**Fig 2:**
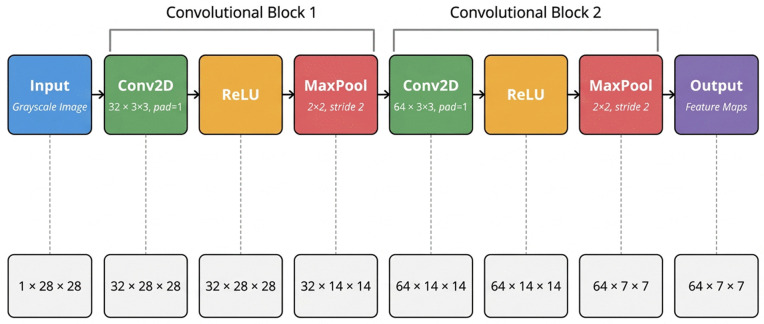
CNN Module. Architecture of the CNN Feature Extractor with two convolutional blocks, transforming input image of size [1×28×28] to output feature maps of size [64×7×7].

**Fig 3:**
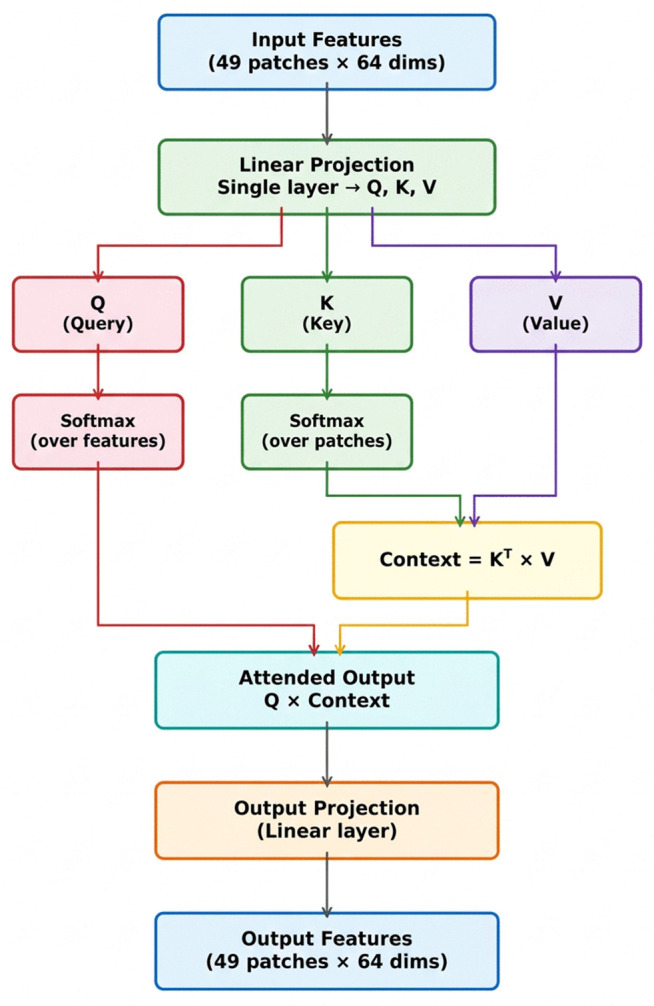
LSA Module. Flowchart of the Linear Self-Attention module performing Q, K, V projections with dual Softmax and context computation, mapping 49 tokens × 64 dimensions to attended output features.

The TinyCNN-LSA model was built with the purpose of reducing computational cost and training time. It consists of three modules: a lightweight convolutional feature extractor for spatial pattern encoding, a multi-head linear self-attention (LSA) encoder to capture global dependencies efficiently, and a fully connected classifier for final decision making. Fig 1 is the overall architecture of the proposed model. Figs 2 and 3 represents the CNN and LSA modules respectively.

### Input representation and data flow initialization

Each 28×28 grayscale image was converted into a 3D tensor with shape [1,28]. After being grouped into a batch, the shape became [*B*, 1, 28, 28], where *B* is the batch size. The values were normalized and converted to floating-point tensors. The single channel reflects the monochrome nature of the Fashion MNIST dataset.

### Convolutional feature extractor

The convolutional feature extractor consists of two main blocks designed to learn increasingly abstract visual features from the input images. To extract the basic patterns, edges and textures from images, the first block contains a 2D convolutional layer with 32 learnable filters of size 3×3. This results in feature maps. Non-linearity is introduced in the feature maps through the ReLU activation function. Then a 2×2 max-pooling operation is applied to reduce the spatial dimensions by selecting the maximum value from each patch. This helps to retain the most prominent features. The next step is mid-level feature formation. By using the same structure of convolution, ReLU, and max-pooling, the second convolutional block performs mid-level feature formation. This block captures more abstract patterns by building on the outputs of the previous block. The resulting output tensor has the shape [*B*, 64, 7, 7]. This makes it suitable for spatial tokenization to be performed in the next stage of the model. The mathematical operations of both convolutional blocks are described as follows:

#### First convolutional block.

Let the input image tensor be represented by $𝐗\in {\mathbb{R}}^{B\times 1\times 28\times 28}$, where *B* is the batch size, 1 indicates the number of input channels, and 28×28 is the spatial resolution of each input image. The first convolutional block applies 32 filters of size 3×3 with a padding of 1. This results in an output tensor with the same spatial resolution but 32 channels. ${𝐖}^{\left(1\right)}\in {\mathbb{R}}^{32\times 1\times 3\times 3}$ denotes the the weights of this block, and ${𝐛}^{\left(1\right)}\in {\mathbb{R}}^{32}$ represents the biases. The output of the convolution for batch index n∈{1,…,B}, output channel c∈{1,…,32}, and spatial location (*i*, *j*) is given by:


$$\begin{array}{c} z_{n,c,i,j}^{\left(1\right)}=\sum_{k=0}^{2}\sum_{l=0}^{2}{𝐗}_{n,0,i+k-1,j+l-1}\;{𝐖}_{c,0,k,l}^{\left(1\right)}+b_{c}^{\left(1\right)} \end{array}$$ (1)

Where $z_{n,c,i,j}^{\left(1\right)}$ is the pre-activation at batch sample *n*, output channel *c*, and spatial position (*i*,*j*); n=1,…,B; c=1,…,32; i,j=1,…,28; and *k* = 0,1,2 indexes the kernel row of the 3×3 filter.

The next step is ReLU activation:


$$\begin{array}{c} a_{n,c,i,j}^{\left(1\right)}=\max(0,\;z_{n,c,i,j}^{\left(1\right)}) \end{array}$$ (2)

Where $a_{n,c,i,j}^{\left(1\right)}$ is the ReLU activation output of the first convolutional block.

The spatial dimensions are reduced from 28×28 to 14×14 by applying a 2×2 max pooling operation with a stride of 2:


$$\begin{array}{c} a_{n,c,i,j}^{\prime \left(1\right)}=\max\{\;a_{n,c,2i+m,2j+n}^{\left(1\right)}\;|\;m,n\in \{0,1\}\} \end{array}$$ (3)

Where $a_{n,c,i,j}^{\prime \left(1\right)}$ is the pooled output of the first convolutional block, and m,n∈{0,1} index the row and column within the 2×2 pooling window. Let the output of the first convolutional block is denoted by ${𝐀}^{\left(1\right)}\in {\mathbb{R}}^{B\times 32\times 14\times 14}$.


$$\begin{array}{c} {}[{𝐀}^{\left(1\right)}{]}_{n,c,i,j}=a_{n,c,i,j}^{\prime \left(1\right)} \end{array}$$ (4)

#### Second convolutional block.

**A**^(1)^ is given as input to the second convolutional block. 64 filters of size 3×3 are applied on the input with padding 1. ${𝐖}^{\left(2\right)}\in {\mathbb{R}}^{64\times 32\times 3\times 3}$ denotes the weights and ${𝐛}^{\left(2\right)}\in {\mathbb{R}}^{64}$ represents the biases. The output of this block is:


$$\begin{array}{c} z_{n,c,i,j}^{\left(2\right)}=\sum_{d=1}^{32}\sum_{k=0}^{2}\sum_{l=0}^{2}a_{n,d,i+k-1,j+l-1}^{\prime \left(1\right)}\;{𝐖}_{c,d,k,l}^{\left(2\right)}+b_{c}^{\left(2\right)} \end{array}$$ (5)

Where $z_{n,c,i,j}^{\left(2\right)}$ is the pre-activation output of the second convolutional block, and d=1,…,32 indexes the input channels from the previous block.

Next step is ReLU activation:


$$\begin{array}{c} a_{n,c,i,j}^{\left(2\right)}=\max(0,\;z_{n,c,i,j}^{\left(2\right)}) \end{array}$$ (6)

Where $a_{n,c,i,j}^{\left(2\right)}$ is the ReLU activation output of the second convolutional block.

A 2×2 max pooling operation is performed:


$$\begin{array}{c} a_{n,c,i,j}^{\prime \left(2\right)}=\max\{\;a_{n,c,2i+m,2j+n}^{\left(2\right)}\;|\;m,n\in \{0,1\}\} \end{array}$$ (7)

Where $a_{n,c,i,j}^{\prime \left(2\right)}$ is the pooled output of the second convolutional block. Let ${𝐀}^{\left(2\right)}\in {\mathbb{R}}^{B\times 64\times 7\times 7}$ denote the output tensor of second convolutional block.


$$\begin{array}{c} {}[{𝐀}^{\left(2\right)}{]}_{n,c,i,j}=a_{n,c,i,j}^{\prime \left(2\right)} \end{array}$$ (8)

### Spatial Tokenization and linear self-attention encoder

To prepare the 4D tensor ${𝐀}^{\left(2\right)}\in {\mathbb{R}}^{B\times C\times H\times W}$ for the Linear Self-Attention (LSA) encoder, it is rearranged into a sequence of spatial tokens. The tensor is first permuted to shape ${\mathbb{R}}^{B\times 7\times 7\times 64}$, and then reshaped into a 3D tensor of shape ${𝐗}_{attn}\in {\mathbb{R}}^{B\times N\times C}$, where N=7×7=49 is the number of tokens, *C* = 64 is the embedding dimension of each token, number of heads *h* = 4, and per–head dimension *C*/*h* = 16:


$$\begin{array}{c} {𝐗}_{attn}=Reshape(Permute({𝐀}^{\left(2\right)},(0,2,3,1)))\;\in \;{\mathbb{R}}^{B\times 49\times 64} \end{array}$$ (9)

Where **X**_attn_ is the sequence of N=7×7=49 spatial tokens obtained by permuting the output tensor ${𝐀}^{\left(2\right)}\in {\mathbb{R}}^{B\times 64\times 7\times 7}$ into shape ${\mathbb{R}}^{B\times 7\times 7\times 64}$ and then flattening the 7×7 spatial dimensions into the token dimension, each token having a 64-dimensional feature vector.

#### Query–key–value projection.

The input sequence **X**_attn_ is linearly projected into concatenated queries, keys, and values:


$$\begin{array}{c} QKV={𝐗}_{attn}\;{𝐖}_{qkv}+{𝐛}_{qkv}\;\in \;{\mathbb{R}}^{B\times N\times 3C} \end{array}$$ (10)

Where QKV is the concatenation of queries, keys, and values for each of the *N* tokens per batch; ${𝐖}_{qkv}\in {\mathbb{R}}^{C\times 3C}$ is the combined projection matrix and ${𝐛}_{qkv}\in {\mathbb{R}}^{3C}$ is its bias.

#### Softmax normalization.

Rather than computing full $QK^{⊤}$, linear self-attention applies separable softmax:


$$\begin{array}{cc} \tilde{𝐐} & =Softmax(𝐐,\;dim=-1)\;(\text{over features, }C/h=16),\\ \tilde{𝐊} & =Softmax(𝐊,\;dim=-2)\;(\text{over tokens, }N=49). \end{array}$$

$$\begin{array}{c} LinearAttn(Q,K,V)=\tilde{𝐐}\;({\tilde{𝐊}}^{T}\;𝐕) \end{array}$$ (11)

Where LinearAttn(Q,K,V) is the linear self‐attention output tensor of shape ${\mathbb{R}}^{B\times h\times N\times (C/h)}$; $\tilde{𝐐}\in {\mathbb{R}}^{B\times h\times N\times (C/h)}$ are the feature‐wise normalized queries, $\tilde{𝐊}\in {\mathbb{R}}^{B\times h\times N\times (C/h)}$ are the token‐wise normalized keys, and $𝐕\in {\mathbb{R}}^{B\times h\times N\times (C/h)}$ are the values.

#### Context matrix.

The context for each head is


$$\begin{array}{c} {𝐂}_{b,h,:,:}=\sum_{n=1}^{N}{\tilde{𝐊}}_{b,h,n,:}\;⊗\;{𝐕}_{b,h,n,:},\;𝐂\in {\mathbb{R}}^{B\times h\times (C/h)\times (C/h)}. \end{array}$$ (12)

Where ${𝐂}_{b,h,:,:}$ is the context matrix for batch *b* and head *h*; b=1,…,B; h=1,…,4; and n=1,…,N indexes the tokens over which keys and values are contracted.

#### Attention output.

For each token,


$$\begin{array}{c} {𝐙}_{b,h,n,:}={\tilde{𝐐}}_{b,h,n,:}\;{𝐂}_{b,h,:,:} \end{array}$$ (13)

Where ${𝐙}_{b,h,n,:}$ is the attended output.

#### Merge heads and output projection.

The multi‐head outputs $𝐙\in {\mathbb{R}}^{B\times h\times N\times (C/h)}$ are merged and projected:


$$\begin{array}{c} {𝐙}_{merged}=Reshape(𝐙)\;\cdot \;{𝐖}_{proj}+{𝐛}_{proj}\;\in \;{\mathbb{R}}^{B\times N\times C} \end{array}$$ (14)

Where **Z**_merged_ is the merged output sequence of *N* tokens per batch after combining the *h* attention heads and reshaping to ${\mathbb{R}}^{B\times N\times C}$, ${𝐖}_{proj}\in {\mathbb{R}}^{C\times C}$ is the output projection matrix, and ${𝐛}_{proj}\in {\mathbb{R}}^{C}$ is its bias.

#### Fully connected classifier (multi layer perceptron head).

The token sequence is flattened into a vector per image for the MLP:


$$\begin{array}{cc} {𝐙}_{flat} & =Flatten({𝐙}_{merged},\;start\_dim=1),\\ & \in {\mathbb{R}}^{B\times (N\cdot C)}={\mathbb{R}}^{B\times 3136}\;. \end{array}$$ (15)

Where **Z**_flat_ is the final flattened representation for each image, obtained by concatenating *N* tokens of dimension *C* into a single vector per batch sample. *N* = 49, and *C* = 64 so N·C=49×64=3136. The fully connected classifier (MLP Head) is the final decision-making step of the neural network. MLP Head translates the learned features from earlier layers into class predictions. It takes as input the flattened vector $Z_{\text{flat}}\in {\mathbb{R}}^{B\times 3136}$.

#### Linear projection to hidden dimension.

To project **Z**_flat_ into a lower-dimensional hidden space, it is passed through a fully connected linear layer. In this case, the hidden dimension is *H*_1_ = 128. This transformation is defined as:


$$\begin{array}{c} {𝐡}_{1}={𝐙}_{flat}\;{𝐖}_{1}+{𝐛}_{1},\;{𝐖}_{1}\in {\mathbb{R}}^{3136\times 128},\;{𝐛}_{1}\in {\mathbb{R}}^{128} \end{array}$$ (16)

where **W**_1_ is the learned weight matrix for the first linear layer projecting to a 128-dimensional hidden space, **b**_1_ is the bias vector added to each projected output. The result is an intermediate representation ${𝐡}_{1}\in {\mathbb{R}}^{B\times 128}$, where each image in the batch is now embedded into a 128-dimensional vector.


$$\begin{array}{c} {𝐖}_{1}=[\begin{array}{cccc} w_{1,1}^{\left(1\right)} & w_{1,2}^{\left(1\right)} & \cdots & w_{1,128}^{\left(1\right)}\\ w_{2,1}^{\left(1\right)} & w_{2,2}^{\left(1\right)} & \cdots & w_{2,128}^{\left(1\right)}\\ \vdots & \vdots & \ddots & \vdots \\ w_{3136,1}^{\left(1\right)} & w_{3136,2}^{\left(1\right)} & \cdots & w_{3136,128}^{\left(1\right)} \end{array}]\;\in \;{\mathbb{R}}^{3136\times 128} \end{array}$$ (17)

$$\begin{array}{c} {𝐛}_{1}=[b_{1}^{\left(1\right)},\;b_{1}^{\left(2\right)},\;\ldots ,\;b_{1}^{\left(128\right)}]\;\in \;{\mathbb{R}}^{128} \end{array}$$ (18)

#### ReLU activation.

ReLU activation function is applied to **h**_1_ to introduce non-linearity. This allows the network to capture more complex patterns as compared to a linear transformation alone would do. The result is the activated hidden representation:


$$\begin{array}{c} {𝐡}_{1}^{act}=ReLU({𝐡}_{1}),\;{𝐡}_{1}^{act}\in {\mathbb{R}}^{B\times 128} \end{array}$$ (19)

where ${𝐡}_{1}^{\text{act}}$ is a matrix of shape B×128 where each row is a 128-dimensional activated hidden feature vector for a sample in the batch after applying ReLU.


$$\begin{array}{c} {𝐡}_{1}^{act}=[\begin{array}{cccc} h_{1,1}^{act} & h_{1,2}^{act} & \cdots & h_{1,128}^{act}\\ h_{2,1}^{act} & h_{2,2}^{act} & \cdots & h_{2,128}^{act}\\ \vdots & \vdots & \ddots & \vdots \\ h_{B,1}^{act} & h_{B,2}^{act} & \cdots & h_{B,128}^{act} \end{array}]\;\in \;{\mathbb{R}}^{B\times 128} \end{array}$$ (20)

#### Dropout regularization.

To prevent overfitting, the activated vector ${𝐡}_{1}^{\text{act}}$ is passed through a dropout layer. A random subset of values is set to zero with probability *p* = 0.3:


$$\begin{array}{c} {𝐡}_{1}^{drop}=Dropout({𝐡}_{1}^{act}),\;{𝐡}_{1}^{drop}\in {\mathbb{R}}^{B\times 128} \end{array}$$ (21)

where ${𝐡}_{1}^{\text{drop}}$ is a matrix of shape B×128 and each row is a 128-dimensional hidden feature vector for a sample in the batch after applying dropout regularization.


$$\begin{array}{c} {𝐡}_{1}^{drop}=[\begin{array}{cccc} h_{1,1}^{drop} & h_{1,2}^{drop} & \cdots & h_{1,128}^{drop}\\ h_{2,1}^{drop} & h_{2,2}^{drop} & \cdots & h_{2,128}^{drop}\\ \vdots & \vdots & \ddots & \vdots \\ h_{B,1}^{drop} & h_{B,2}^{drop} & \cdots & h_{B,128}^{drop} \end{array}]\;\in \;{\mathbb{R}}^{B\times 128} \end{array}$$ (22)

#### Final linear layer for classification.

The dropout-regularized hidden vector is passed through a final linear transformation. This maps it to the number of target classes. For a 10-class classification task:


$$\begin{array}{c} 𝐲={𝐡}_{1}^{drop}\;{𝐖}_{2}+{𝐛}_{2},\;{𝐖}_{2}\in {\mathbb{R}}^{128\times 10},\;{𝐛}_{2}\in {\mathbb{R}}^{10} \end{array}$$ (23)

Where $𝐲\in {\mathbb{R}}^{B\times 10}$ is the vector of class logits for each sample in the batch; **W**_2_ is the weight matrix mapping 128 features to 10 classes; and **b**_2_ is the bias vector for the final linear layer.


$$\begin{array}{c} 𝐲=[\begin{array}{cccc} y_{1,1} & y_{1,2} & \cdots & y_{1,10}\\ y_{2,1} & y_{2,2} & \cdots & y_{2,10}\\ \vdots & \vdots & \ddots & \vdots \\ y_{B,1} & y_{B,2} & \cdots & y_{B,10} \end{array}]\;\in \;{\mathbb{R}}^{B\times 10} \end{array}$$ (24)

$$\begin{array}{c} {𝐖}_{2}=[\begin{array}{cccc} w_{1,1} & w_{1,2} & \cdots & w_{1,10}\\ w_{2,1} & w_{2,2} & \cdots & w_{2,10}\\ \vdots & \vdots & \ddots & \vdots \\ w_{128,1} & w_{128,2} & \cdots & w_{128,10} \end{array}]\;\in \;{\mathbb{R}}^{128\times 10} \end{array}$$ (25)

$$\begin{array}{c} {𝐛}_{2}=[\begin{array}{cccc} b_{1} & b_{2} & \cdots & b_{10} \end{array}]\;\in \;{\mathbb{R}}^{10} \end{array}$$ (26)

#### Softmax for probability output during inference.

Softmax function is applied during inference to the logits to obtain class probabilities:


$$\begin{array}{c} 𝐩=Softmax(𝐲),\;𝐩\in {\mathbb{R}}^{B\times 10} \end{array}$$ (27)

$$\begin{array}{c} 𝐩=[\begin{array}{cccc} p_{1,1} & p_{1,2} & \cdots & p_{1,10}\\ p_{2,1} & p_{2,2} & \cdots & p_{2,10}\\ \vdots & \vdots & \ddots & \vdots \\ p_{B,1} & p_{B,2} & \cdots & p_{B,10} \end{array}]\;\in \;{\mathbb{R}}^{B\times 10} \end{array}$$ (28)

$$\begin{array}{c} \sum_{j=1}^{10}p_{i,j}=1,\;\forall \;i=1,\ldots ,B \end{array}$$ (29)

where **p** is the matrix of class probabilities for each sample in the batch after applying the Softmax function, with each row summing to 1 and representing a valid probability distribution over the 10 classes. The CrossEntropyLoss function is used to compare probability distributions with the true labels. It combines the Softmax and Negative Log-Likelihood operations in a numerically stable manner. The model’s classification error over the batch is represented by the loss computed from these comparisons. Table 1 shows the transformations from input to output performed by the TinyCNN-LSA model.

**Table 1:** Data Transformations.

Step	Operation	Input Shape	Output Shape
**CNN Feature Extractor**			
1	Input image	[B, 1, 28, 28]	[B, 1, 28, 28]
2	Conv2d	[B, 1, 28, 28]	[B, 32, 28, 28]
3	ReLU	[B, 32, 28, 28]	[B, 32, 28, 28]
4	MaxPool2d	[B, 32, 28, 28]	[B, 32, 14, 14]
5	Conv2d	[B, 32, 14, 14]	[B, 64, 14, 14]
6	ReLU	[B, 64, 14, 14]	[B, 64, 14, 14]
7	MaxPool2d	[B, 64, 14, 14]	[B, 64, 7, 7]
**Spatial Tokenization**			
8	Permute	[B, 64, 7, 7]	[B, 7, 7, 64]
9	Flatten patches	[B, 7, 7, 64]	[B, 49, 64]
**Linear Self-Attention Encoder**			
10	QKV projection	[B, 49, 64]	[B, 49, 192]
11	Reshape for heads	[B, 49, 192]	[B, 49, 3, 4, 16]
12	Permute	[B, 49, 3, 4, 16]	[3, B, 4, 49, 16]
13	Split into Q, K, V	[3, B, 4, 49, 16]	3×[B, 4, 49, 16]
14	Softmax on Q & K	[B, 4, 49, 16]	[B, 4, 49, 16]
15	Compute context	Q, K, V	[B, 4, 16, 16]
16	Apply linear attention	Q, context	[B, 4, 49, 16]
17	Merge heads (reshape)	[B, 4, 49, 16]	[B, 49, 64]
18	Output projection	[B, 49, 64]	[B, 49, 64]
**Classifier Head**			
19	Flatten	[B, 49, 64]	[B, 3136]
20	Dense projection	[B, 3136]	[B, 128]
21	ReLU	[B, 128]	[B, 128]
22	Dropout regularization	[B, 128]	[B, 128]
23	Logits projection	[B, 128]	[B, 10]

**Note:** B denotes batch size. Shapes are expressed as tensor dimensions throughout the processing pipeline.

### Training configuration

The model was trained using the Cross-Entropy Loss function, which is suitable for multi-class classification problems. The Adam optimizer was utilized with a learning rate of 1e-3, ensuring adaptive and stable updates to model weights. During training, a batch size of 64 was used, while batch size 128 was adopted for validation and testing to optimize memory utilization. The training ran for a maximum of 50 epochs, with early stopping enabled based on validation accuracy, using a patience of 5 epochs to stop training if no improvement was observed. Early Stopping Criterion: In order to prevent the model from overfitting and to minimize unnecessary computation, a manual early stopping method was implemented on the basis of validation accuracy. Following each epoch, the validation accuracy was reviewed against the highest recorded value to decide whether further training should proceed or not. The training process monitored validation accuracy after each epoch. If a new best accuracy was detected, model parameters were saved, and the patience counter was restarted. Without improvement, the patience counter increased. After passing 5 epochs when no further improvement was seen, training was stopped to avoid overfitting. The experiment ran on Google Colab free version, 2-core CPU with 13.61 GB RAM. Peak memory usage was around 1.58 GB. The system was running on Python 3.12.13 and Linux 6.6.113 + . Table 2 presents the training hyperparameters used for the TinyCNN-LSA model. Table 3 details the number of learnable parameters in different layers of the model, which counts to a total of 438,282 parameters.

**Table 2:** Key hyperparameters and settings used to train the model.

Parameter	Value
Loss Function	Cross-Entropy Loss (Softmax)
Optimizer	Adam
Learning Rate	$1\times 10^{-3}$
Batch Size (Train)	64
Batch Size (Val/Test)	128
Max Epochs	50
Early Stopping Patience	5
Dropout Rate	0.3 (in MLP head)

**Table 3:** Parameters Count.

Layer	# Parameters
Conv2d Layer 1	320
Conv2d Layer 2	18,496
Self-Attention QKV Projection	12,480
Self-Attention Output Projection	4,160
Fully Connected Layer 1	401,536
Fully Connected Layer 2	1,290
**Total**	**438,282**

## XAI analysis

### Linear self-attention visualization for spatial interpretability

Understanding what a deep learning model attends to during inference is one of the challenges of Explainable Artificial Intelligence (XAI). This study adopts a post-hoc intrinsic interpretability approach by directly interrogating the internal attention mechanism of the proposed TinyCNN-LSA model, a hybrid architecture that couples a convolutional neural network (CNN) backbone with a Linear Self-Attention (LSA) module. Unlike gradient-based or perturbation-based XAI methods that operate externally to the model, the LSA mechanism is structurally embedded within the forward pass, making its attention weights a natural and computationally appropriate window into the model’s reasoning process. The attention weights, therefore, do not merely approximate model behavior, rather they appear to be a direct product of it, lending this approach a degree of mechanistic transparency that surrogate-based methods may not readily offer. The LSA module operates on the spatial feature map produced by the CNN backbone. After two convolutional blocks with max-pooling, the input image of size 28×28 is reduced to a 7×7 spatial grid with 64 feature channels, yielding 49 spatial tokens each of dimensionality 64. These tokens are passed through the LSA layer, which projects them into query (*Q*), key (*K*), and value (*V*) matrices via a single linear projection. Notably, the LSA formulation applies softmax along the feature dimension of *Q* and softmax along the token dimension of *K*, rather than the conventional scaled dot-product attention. This appears to yield linear complexity with respect to the number of tokens and, more importantly for interpretability, produces a well-defined context matrix via the einsum $K^{⊤}V$, subsequently combined with *Q* to produce attended outputs. To extract the attention matrix for visualization, the pairwise token-to-token attention scores are computed as $A=Q^{⊤}K^{⊤}$, averaged across the four attention heads, yielding a 49×49 matrix where entry *A*[*i*,*j*] represents the degree to which spatial token *i* attends to spatial token *j* across the 7×7 feature grid.

The visualization pipeline proceeds through several well-defined stages. For each of the ten Fashion MNIST classes, one representative test sample is selected. The CNN backbone of the proposed model is applied to extract the 7×7×64 feature map, which is reshaped into a 49×64 token sequence and passed through the LSA layer. The resulting 49×49 attention matrix *A* is computed by averaging over all four attention heads, a choice that appears appropriate for ensuring a stable, class-representative signal rather than a potentially noisy single-head view. Two distinct spatial signals are then derived from *A*: (1) the Attention Received Map, computed as the column-wise mean of *A*, representing how much aggregate attention each spatial token receives from all other tokens, and (2) the Query-Specific Attention Map, extracted as the row corresponding to the most-attended token, revealing which spatial locations that dominant token appears to focus on. Both maps are normalized independently to [0, 1] per sample to ensure visual comparability across classes with varying feature scales. The resulting 7×7 maps are subsequently bilinearly upsampled to the original 28×28 resolution for spatial overlay onto the input image.

**Fig 4:**
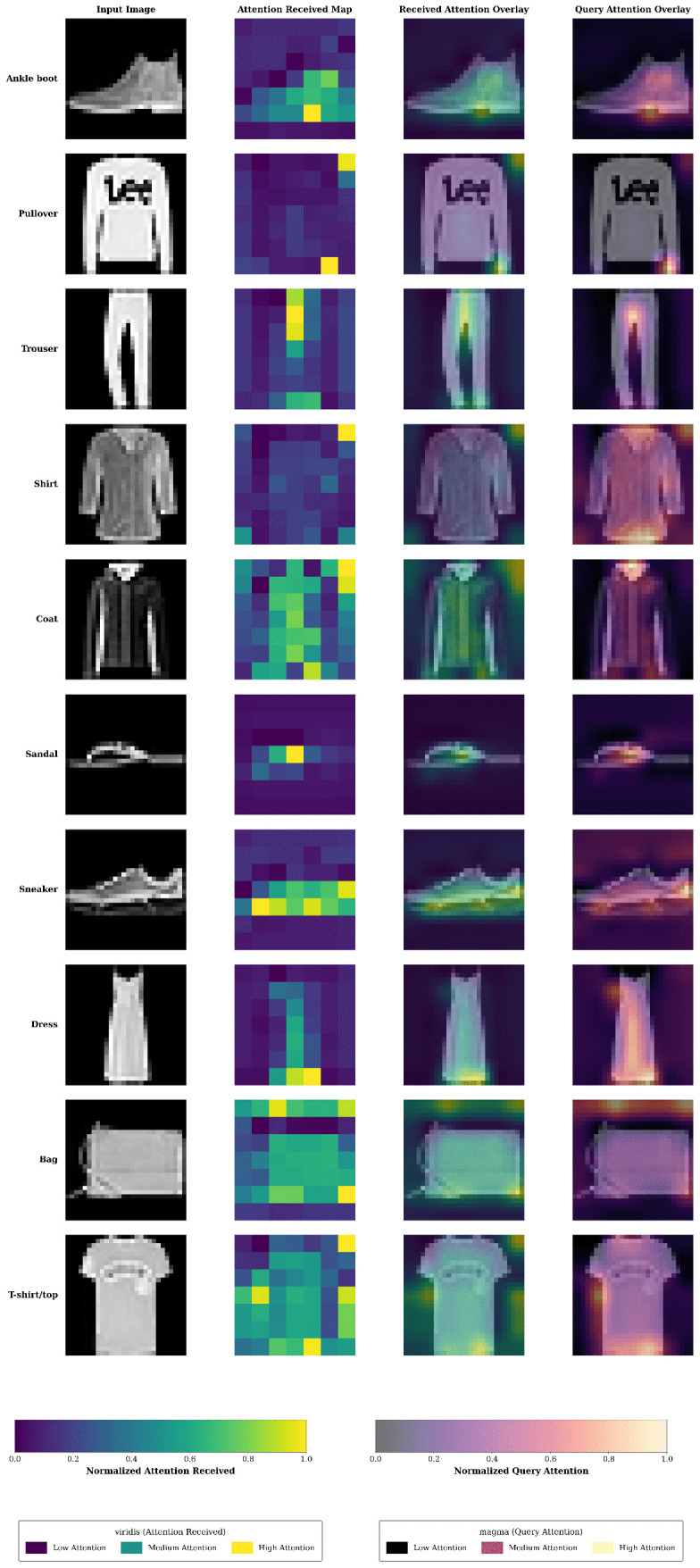
Linear Self-Attention visualization across all ten Fashion MNIST classes. Each row shows the input image, attention received map (viridis colormap), received attention overlay, and query attention overlay (magma colormap).

The resulting visualization, presented in Fig 4, is organized as a 10×4 grid where each row corresponds to one Fashion MNIST class with the same true label and predicted label annotated alongside the original grayscale input in Column 1. Column 2 presents the raw 7×7 Attention Received Map rendered in the *viridis* colormap, where deep purple and blue encode low attention and bright yellow encodes high attention, with all values normalized per sample to [0, 1] and the colorbar on the bottom encoding this normalized intensity from 0.0 to 1.0. Column 3 overlays the bilinearly upsampled Attention Received Map (*viridis*, transparency-α=0.55) onto the input image, allowing direct spatial alignment between attended regions and visible image content. Column 4 overlays the Query-Specific Attention Map in the *magma* colormap (transparency-α=0.55), where black and dark purple represent negligible attention and bright pink and white represent the highest focused attention from the dominant query token, offering a complementary spatial perspective on the model’s attentional behavior across all ten classes.

Examining the visualization across all ten classes reveals several patterns that may speak to the model’s learned representational strategy. For structurally compact objects such as the Sandal and Ankle Boot, the attention appears sharply concentrated on the object’s sole and toe-box region, the most geometrically distinctive areas that may differentiate footwear categories from one another. The bright yellow patches in Column 2 for these classes are spatially localized to approximately 2–4 tokens out of the 49 available, suggesting a highly sparse and potentially discriminative attention pattern. In contrast, garments with large, uniform texture regions such as the Pullover and Coat exhibit a more distributed attention profile, with moderate-intensity teal and green hues spread across the torso region, consistent with the possibility that the model integrates global shape information rather than anchoring on a single salient keypoint. The Trouser class presents a particularly informative case, as the Attention Received Map reveals a narrow vertical band of relatively high attention along the central seam of the trousers, which may correspond to the most structurally distinctive feature of this elongated, bilaterally symmetric garment. The Dress class similarly shows concentrated attention around the upper shoulder and strap region, which appears to be the primary visual cue that distinguishes a dress from a coat or pullover at the global shape level.

The Query-Specific Attention Maps in Column 4 offer a complementary and potentially more fine-grained interpretive perspective. Rendered in the *magma* colormap, these maps isolate the attentional field-of-view of the single most-attended spatial token, effectively posing the question of which spatial locations that dominant token appears to focus on. For the T-Shirt/Top and Shirt classes, the *magma* overlay highlights the collar and shoulder regions with warm pink intensities, suggesting that the model’s dominant token may anchor itself to the neckline, a region that is visually consistent across both classes but differs subtly in shape. This observation may partly account for inter-class confusion that could be observed in broader classification evaluations. For the Bag class, the *magma* map distributes attention broadly across the rectangular body of the bag, which may reflect the relatively featureless and uniform texture that appears to demand holistic spatial integration rather than local keypoint detection. For the Sneaker class, a concentrated warm region over the midsole and toe cap area complements the received attention map, suggesting that the model consistently attends to the lower half of the footwear silhouette to distinguish sneakers from sandals and ankle boots. Across all ten selected samples, every instance was correctly classified, as the true label (T:) matches the predicted label (P:) in all rows of Fig 4, and this outcome is visually supported by the attention maps, which appear to consistently highlight semantically meaningful and class-relevant regions rather than background or spatially irrelevant positions.

The potential utility of this XAI approach is both methodological and practical. From a methodological standpoint, the direct extraction of attention weights from the model’s built-in attention mechanism may avoid the approximation errors that are more commonly associated with post-hoc gradient or occlusion-based methods, suggesting that the explanations could be more faithful to the model’s actual decision process. From a practical standpoint, the overlay visualizations appear immediately interpretable by domain practitioners without requiring statistical expertise, as the color gradient from dark to bright directly encodes the model’s spatial priority, and the four-column layout enables rapid cross-class comparison of attentional strategies. The choice of bilinear upsampling from 7×7 to 28×28 introduces a degree of spatial smoothing that, while not pixel-precise, appears to produce visually coherent heatmaps that respect object boundaries and remain broadly faithful to the coarse spatial resolution of the feature map. One limitation could be that the LSA formulation, by applying separate softmax operations along the feature and token dimensions of *Q* and *K* respectively, rather than the joint normalized dot-product of standard attention, produces attention scores that may not be directly probabilistic in the conventional sense. However, the per-sample normalization applied in this visualization pipeline appears to ensure that the visual encoding remains consistent and interpretable across all classes, making this an appropriate and computationally efficient approach to attention-based explainability in hybrid CNN-attention architectures trained on fashion image recognition tasks.

### Per-head attention analysis across spatial scales

One of the structural advantages of multi-head attention mechanisms is that individual heads may learn to specialize in capturing distinct spatial relationships, a property that becomes particularly valuable from an interpretability standpoint. This study extends the attention visualization analysis by decomposing the LSA module into its constituent heads and examining the attention received maps produced by each head independently, rather than relying solely on the head-averaged signal explored in the preceding section. This per-head decomposition offers a potentially more granular window into the spatial reasoning strategies that emerge across the four attention heads of the TinyCNN-LSA architecture, and may reveal whether different heads specialize in attending to different spatial regions of the input, or whether their attentional patterns remain broadly redundant.

The implementation follows a procedure closely related to that of the averaged attention visualization, with one key distinction. Rather than averaging the attention matrix *A* across all four heads prior to spatial analysis, the per-head attention matrices are retained separately. For each head *h*, the attention received map is computed as the column-wise mean of the head-specific 49×49 attention matrix $A_{h}$, yielding a 7×7 spatial map that encodes how much aggregate attention each spatial token receives from all other tokens within that particular head. Given that the model contains four attention heads in total, this study visualizes the first three heads, denoted Head 1, Head 2, and Head 3, as a representative subset that appears sufficient for identifying patterns of head-level specialization. The per-head maps are jointly normalized across all heads for a given sample, with the minimum and maximum values computed over the full stack of head maps, so that the color encoding remains directly comparable across heads within the same row. Each 7×7 map is subsequently bilinearly upsampled to 28×28 for spatial overlay visualization.

**Fig 5:**
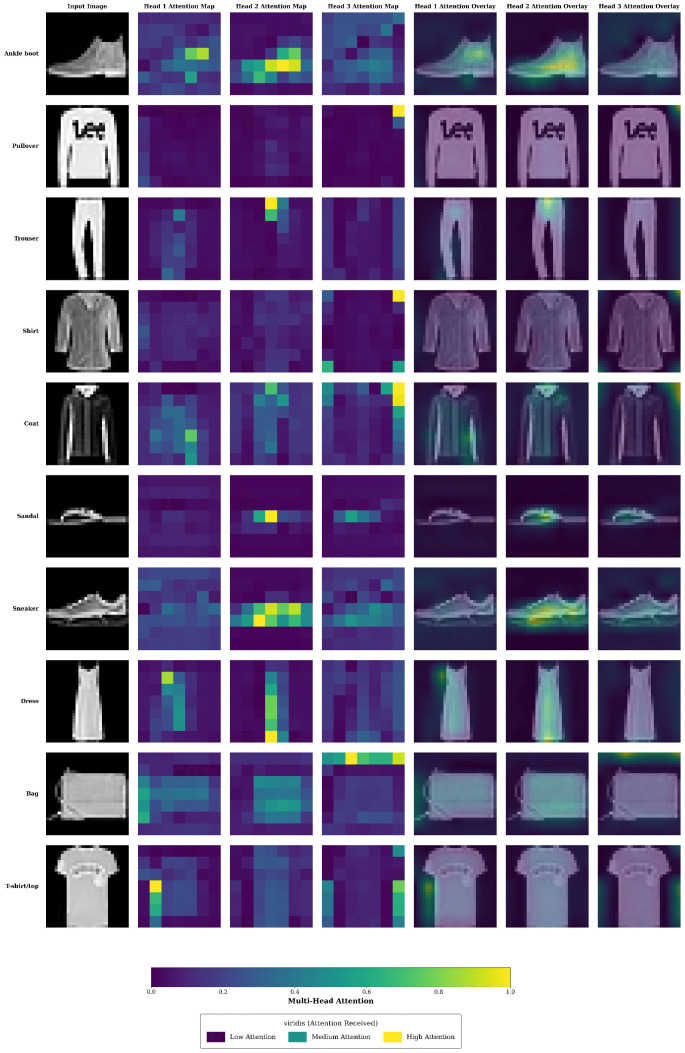
Per-head Linear Self-Attention visualization across all ten Fashion MNIST classes. Each row displays the input image alongside attention maps and overlays for Heads 1, 2, and 3 (viridis colormap).

The visualization, presented in Fig 5, is organized as a 10×7 grid where each row corresponds to one correctly classified Fashion MNIST class. The first column shows the original grayscale input image. Columns 2–4 display the raw 7×7 Attention Received Maps for Head 1, Head 2, and Head 3 respectively, rendered in the *viridis* colormap where deep purple and blue encode low attention and bright yellow encodes high attention. Columns 5–7 display the corresponding 28×28 bilinearly upsampled overlays of each head’s attention map onto the input image at a transparency of α=0.55, allowing direct spatial alignment between attended regions and visible image structure.

Examining the per-head maps across all ten classes suggests that the three visualized heads exhibit meaningfully different spatial behaviors, a finding that may support the notion of functional specialization within the multi-head attention mechanism. For the Ankle Boot class, Head 1 appears to distribute attention broadly across the lower half of the feature grid, whereas Head 2 concentrates a sharp, localized high-attention region toward the sole and heel area, and Head 3 produces a more diffuse pattern that spans a larger portion of the spatial grid. This divergence across heads for the same input suggests that individual heads may be encoding complementary spatial cues rather than redundant ones, which could contribute to the robustness of the model’s classification decision. For the Trouser class, Head 2 produces a notably concentrated vertical band of high attention along the central region of the garment, consistent with the seam-like structural feature identified in the averaged attention analysis, while Head 1 and Head 3 show comparatively lower and more spatially dispersed attention, suggesting that the discriminative signal for this class may be predominantly captured by a single head.

The footwear classes, particularly Sandal and Sneaker, present some of the most visually informative per-head patterns in the figure. For the Sandal class, all three heads appear to concentrate attention on the horizontal mid-region of the image where the sole of the sandal is located, though the precise spatial peak differs slightly across heads. Head 2 in particular produces the sharpest and most spatially confined high-attention region for this class, suggesting it may be the most discriminatively active head for compact, horizontally oriented objects. For the Sneaker class, Head 2 again produces a prominent bright region over the midsole area, while Head 3 distributes attention more broadly across the upper portion of the shoe, potentially capturing the tongue and lace region as a complementary structural cue. These observations, while qualitative in nature, are consistent with the hypothesis that different heads may attend to different parts of the same object, collectively providing a richer spatial representation than any single head could offer independently.

For garment classes such as the Pullover, Shirt, and Coat, the per-head maps reveal a pattern that may be characterized as globally distributed attention with head-specific local emphases. Head 3 for the Pullover class, for instance, produces a notably bright region in the upper corner of the 7×7 map, which when upsampled appears to correspond to the top-right shoulder region of the garment, a structurally distinctive area that may help differentiate pullovers from coats and shirts. The Shirt class similarly shows Head 3 producing a concentrated attention region in the lower portion of the map, potentially corresponding to the hem or lower body of the garment, while Heads 1 and 2 attend more uniformly to the torso. The Bag class presents a visually distinct pattern in which Head 1 produces a bright region along the left edge of the feature map, Head 2 concentrates on the central body, and Head 3 distributes attention more uniformly, collectively suggesting that the model may be encoding the rectangular outline of the bag through spatially distributed but complementary head-level signals.

The per-head overlay maps in Columns 5–7 reinforce and spatially contextualize the patterns observed in the raw maps. The bilinear upsampling preserves the general spatial structure of each head’s attention while smoothing the coarse 7×7 grid into a visually coherent heatmap that aligns with the object boundaries visible in the grayscale input. The use of a consistent *viridis* colormap across both raw and overlay visualizations, combined with joint per-sample normalization across heads, ensures that the relative intensity differences between heads remain interpretable and are not distorted by scale differences. Across all ten classes, the correctly predicted labels confirm that the attentional patterns observed, even when distributed or diffuse, appear sufficient for accurate classification, and the per-head decomposition suggests that this accuracy may arise from the complementary integration of spatially diverse attentional signals across the multiple heads of the LSA module.

### Attention flow mapping from spatial query positions

A limitation of aggregated attention visualization approaches, such as the averaged and per-head received attention maps explored in the preceding sections, is that they collapse the directional structure of the attention matrix into a single spatial summary, potentially obscuring the relational nature of attention as a token-to-token communication mechanism. This study addresses this limitation by implementing an attention flow analysis, a visualization strategy that probes the attention matrix from the perspective of specific spatial query positions rather than summarizing attention received across all positions. By selecting a set of fixed spatial tokens as query anchors and visualizing the attention each anchor distributes to all other spatial locations, this approach offers a more granular and directionally explicit account of how spatial information propagates through the LSA module during inference.

The implementation proceeds as follows. For each test sample, the averaged attention matrix *A* of shape 49×49 is computed in the same manner as described previously, by extracting the query and key projections from the LSA layer, applying the appropriate softmax operations, and averaging the resulting pairwise attention scores across all four heads. Rather than summarizing this matrix column-wise, the attention flow analysis extracts individual rows of *A*, where each row *A*[*q*,:] encodes the attention that query token *q* distributes across all 49 spatial key tokens. Five query positions are selected at fractional intervals along the token sequence, corresponding to approximately the 25th, 37.5th, 50th, 62.5th, and 75th percentile positions of the flattened 7×7 spatial grid. These positions are chosen to provide a spatially distributed and representative set of query anchors that span the upper, central, and lower regions of the feature map, rather than concentrating queries in a single area. Each extracted attention row is reshaped into a 7×7 map and normalized to [0, 1] by dividing by its maximum value, ensuring that the attention flow from each query position is visualized on a consistent scale. The query token’s spatial location within the 7×7 grid is computed by converting its linear index to row and column coordinates, and is marked with a red cross marker on the corresponding input image to indicate the precise spatial origin of each attention flow map.

**Fig 6:**
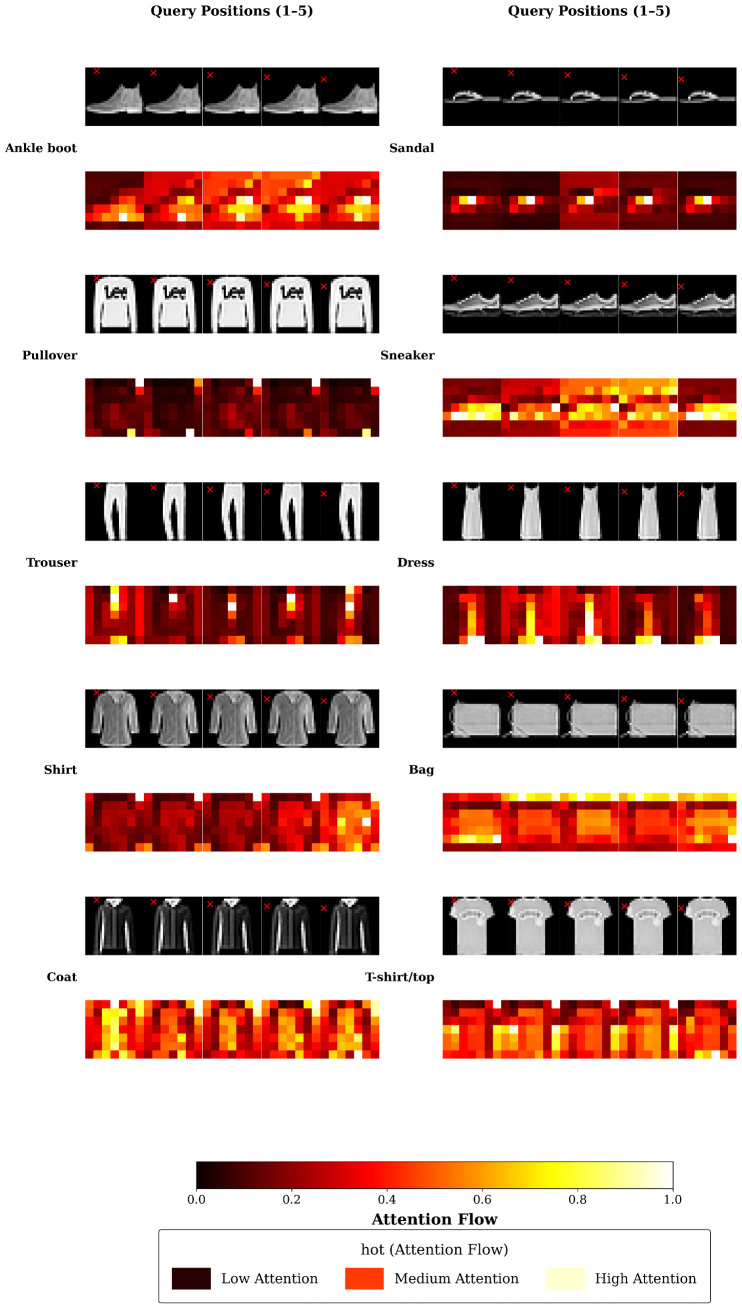
Attention flow maps from five spatial query positions across all ten Fashion MNIST classes. Each class shows five query positions (marked with red ×) and their corresponding attention flow maps (hot colormap).

The visualization, presented in Fig 6, is organized as a 2×5 block layout where the ten correctly classified Fashion MNIST classes are arranged in two column blocks of five classes each. Within each class block, the upper row displays the original grayscale input image five times, once for each query position, with a red cross marker indicating the spatial location of the query token on the 7×7 feature grid. The lower row displays the corresponding attention flow maps rendered in the *hot* colormap, where dark red and black indicate negligible attention, and bright yellow and white indicate the highest attention values.

Examining the attention flow maps across all ten classes and five query positions reveals several patterns that may offer insight into the spatial communication structure of the LSA module. A consistent and notable observation is that attention flow maps tend to exhibit high-intensity regions that are spatially proximate to the query token’s location, suggesting that the LSA module may preferentially attend to local spatial neighborhoods rather than establishing long-range cross-image dependencies. This locality bias appears particularly pronounced for classes with spatially compact and well-defined object boundaries, such as the Ankle Boot and Sandal, where the bright white and yellow regions in the attention flow maps cluster tightly around the query position regardless of which of the five positions is selected. This pattern may suggest that for compact objects, the spatial tokens within the LSA module tend to gather contextual information from their immediate neighbors, a behavior that could be interpreted as a form of local feature refinement rather than global context aggregation.

For garment classes with larger and more spatially extended structures, such as the Trouser and Dress, the attention flow maps reveal a qualitatively different pattern. When the query position is located in the central vertical region of the feature grid, the attention flow appears to extend along the vertical axis of the image, producing elongated bright regions in the hot colormap that align with the structural orientation of the garment. This vertically oriented attention flow may reflect the model’s implicit encoding of the elongated bilateral symmetry of these garments, as the central tokens appear to attend to other tokens along the same vertical axis rather than distributing attention isotropically. For the Pullover and Shirt classes, the attention flow maps suggest a more horizontally distributed pattern when query positions are located in the upper-central region of the grid, potentially corresponding to the shoulder and sleeve area of the garment where horizontal structural extent is most prominent.

The Bag class presents a particularly distinctive attention flow pattern that may be attributed to the rectangular and largely featureless nature of the object. Across all five query positions, the attention flow maps for the Bag class exhibit broadly distributed high attention regions that span a substantial portion of the 7×7 grid, with relatively little spatial concentration around the query position. This diffuse attention flow may suggest that the spatial tokens within the Bag representation do not exhibit strong local preferences, and instead distribute attention more uniformly across the feature map, consistent with the possibility that holistic shape encoding rather than local feature detection is the dominant representational strategy for this class. The Coat class similarly shows a relatively distributed attention flow, though with somewhat higher concentration in the central torso region, which may correspond to the structural boundary between the coat body and the background.

The attention flow visualization, while qualitative in nature, appears to offer a complementary and directionally explicit perspective on the spatial reasoning of the LSA module that aggregated attention maps cannot provide. By anchoring the analysis to specific query positions and tracing the attention each position distributes across the spatial grid, this approach makes the relational structure of the attention mechanism more directly observable, and may support a more nuanced interpretation of how the model integrates spatial context during classification. The use of the *hot* colormap, with its intuitive progression from dark to bright encoding low to high attention, appears appropriate for communicating the directional intensity of attention flow in a visually accessible manner. One consideration worth noting is that the five query positions are selected at fixed fractional intervals rather than adaptively chosen based on class-specific structure, which may result in some query positions falling in spatially uninformative regions such as the background for certain classes.

### Attention rollout for residual-aware spatial saliency

The attention visualization approaches explored in the preceding sections operate directly on the raw attention weights produced by the LSA module, without accounting for the residual connections that are present in transformer-style architectures. In standard attention-based models, residual connections allow information to bypass the attention mechanism entirely, meaning that raw attention weights alone may not fully represent the actual flow of information through the network. Attention Rollout, originally proposed as a method for interpreting vision transformers, addresses this concern by augmenting the attention matrix with an identity term that explicitly models the residual pathway, thereby producing a more complete and arguably more faithful representation of how information propagates through the attention layer. This study applies a single-layer adaptation of Attention Rollout to the TinyCNN-LSA architecture, where the LSA module constitutes the sole attention layer, making the rollout computation a direct and interpretable augmentation of the averaged attention matrix rather than a recursive product across multiple layers.

The implementation proceeds as follows. The averaged attention matrix *A* of shape 49×49 is first computed in the standard manner, by extracting the query and key projections from the LSA layer, applying the appropriate softmax operations along the feature and token dimensions respectively, and averaging the resulting pairwise attention scores across all four attention heads. The Attention Rollout augmentation is then applied by adding the identity matrix *I* of matching dimensions to *A*, yielding the augmented matrix $\hat{A}=A+I$. This addition models the residual connection by ensuring that each spatial token retains a self-attention component, reflecting the possibility that a token’s own representation contributes to its output independently of the attention-weighted context. The augmented matrix $\hat{A}$ is subsequently row-normalized so that each row sums to unity, producing a well-defined row-stochastic matrix that may be interpreted as a transition probability distribution over spatial tokens. To obtain a single global spatial saliency map, the column-wise mean of $\hat{A}$ is computed, yielding a vector of length 49 that represents the aggregate attention received by each spatial token under the residual-augmented attention distribution. This vector is reshaped into a 7×7 map, normalized to [0, 1] by dividing by its maximum value, and bilinearly upsampled to 28×28 for overlay visualization on the original input image.

**Fig 7:**
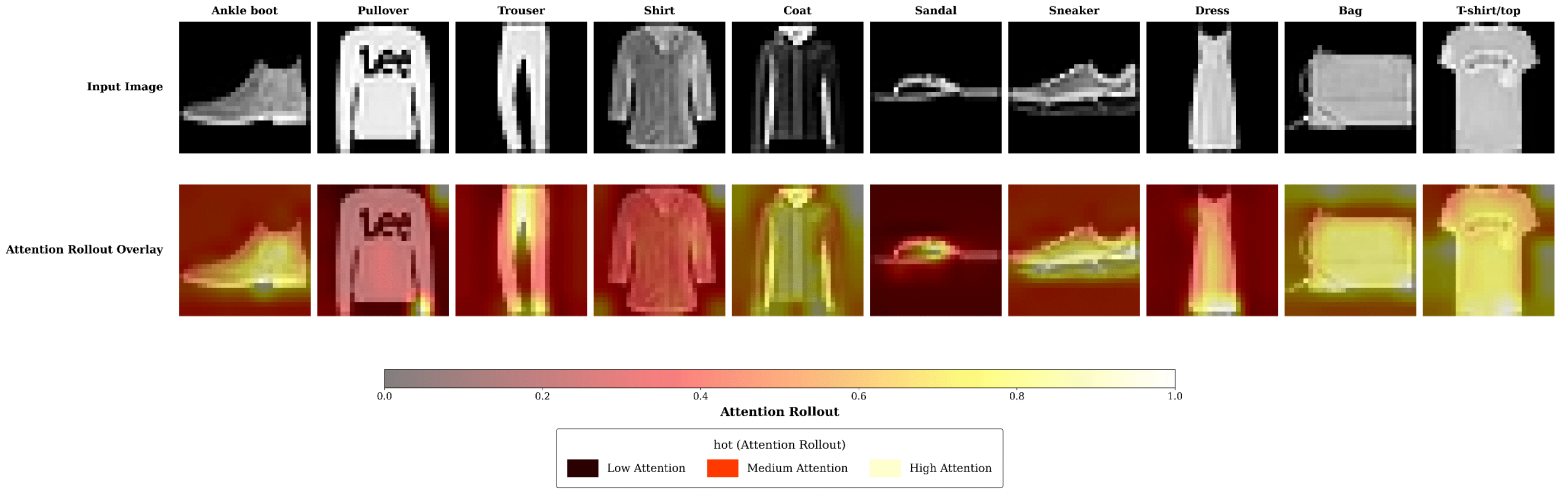
Attention Rollout saliency maps across all ten Fashion MNIST classes. Each column shows the input image and its corresponding attention rollout overlay (hot colormap).

The visualization, presented in Fig 7, is organized as a 2×10 grid where each column corresponds to one correctly classified Fashion MNIST class. The upper row displays the original grayscale input image for each class, and the lower row displays the Attention Rollout saliency map overlaid on the input image using the *hot* colormap at a transparency of α=0.5. In the *hot* colormap, dark red and black encode low rollout attention values and bright yellow and white encode high rollout attention values. The full grayscale input is rendered at full opacity beneath the heatmap overlay, allowing the spatial correspondence between high-attention regions and visible image structure to be assessed directly.

Examining the rollout saliency maps across all ten classes reveals several patterns that may be attributed to the combined effect of the raw attention weights and the residual augmentation. A notable characteristic of the rollout maps, relative to the raw attention visualizations presented in earlier sections, is that the spatial saliency tends to appear somewhat more broadly distributed across the image, with fewer instances of sharply localized single-token peaks. This broadening effect is consistent with the theoretical expectation of Attention Rollout, as the addition of the identity term introduces a uniform self-attention baseline that smooths the attention distribution and reduces the dominance of any single spatial token. Despite this smoothing, the rollout maps retain meaningful spatial structure that appears broadly aligned with the discriminative regions of each class.

For the Ankle Boot class, the rollout map produces a prominent bright yellow region concentrated along the lower portion of the boot, particularly around the sole and toe area, with a warm orange gradient extending upward along the body of the boot. This pattern appears consistent with the raw attention findings and may suggest that the residual augmentation does not substantially alter the spatial priority of the model for compact, well-defined objects. The Sandal class similarly shows a concentrated bright region along the horizontal sole structure of the sandal, with the rollout map producing a narrow band of high saliency that closely follows the object boundary visible in the grayscale input. For the Sneaker class, the rollout map produces a broader high-attention region that spans the midsole and upper body of the shoe, suggesting that the residual-augmented attention distribution integrates a wider spatial context for this class compared to the more compact sandal.

For the garment classes, the rollout maps reveal patterns that may reflect the structural properties of each clothing category. The Trouser class produces a distinctive vertical band of high saliency running along the central axis of the trousers, consistent with the seam region identified in the raw attention analysis, though the rollout map appears to extend this high-attention region more continuously along the full vertical extent of the garment rather than concentrating it at a single spatial position. The Dress class similarly shows a vertically oriented saliency pattern, with bright yellow regions concentrated along the upper body and shoulder straps of the dress, and a warm gradient extending downward along the skirt. The Coat class produces a somewhat more distributed saliency map, with moderate to high attention values spread across the torso and lower body of the garment, and a notably bright region near the collar area that may correspond to the lapel structure.

The Pullover and Shirt classes both produce rollout maps with relatively broad saliency distributions that cover much of the garment area, with warm orange and red tones dominating the central torso region and brighter yellow patches appearing near the shoulders and collar. The similarity between the rollout maps for these two classes may be consistent with the visual similarity between pullovers and shirts at the global shape level, and could partly account for any inter-class confusion that might arise between these categories in broader evaluations. The Bag class presents a notably distinctive rollout pattern, with bright yellow saliency distributed broadly across the rectangular body of the bag and extending to the edges of the object, which may reflect the holistic shape encoding strategy suggested by the attention flow analysis. The T-Shirt/Top class produces a rollout map with high saliency concentrated around the collar and shoulder region, with a warm gradient extending downward across the body of the shirt, appearing consistent with the collar-anchored attentional behavior observed in the raw attention visualizations.

The Attention Rollout approach appears to offer a potentially more complete account of spatial saliency in the TinyCNN-LSA model by incorporating the residual pathway into the attention analysis, and the resulting maps appear broadly consistent with the raw attention visualizations while exhibiting a smoother and more spatially continuous saliency distribution. This consistency across visualization methods may be interpreted as a form of cross-method validation, suggesting that the spatial priorities identified by the raw attention maps are not artifacts of the aggregation strategy but may reflect genuine properties of the model’s learned representations. One consideration worth noting is that the single-layer nature of the TinyCNN-LSA architecture means that the rollout computation does not involve the recursive matrix multiplication across multiple attention layers that characterizes the original Attention Rollout formulation, which may limit the degree to which the residual augmentation alters the resulting saliency maps relative to the raw attention baseline. Nevertheless, the approach appears methodologically appropriate for this architecture and produces visually coherent and interpretable saliency maps that may complement the other attention-based XAI analyses presented in this study.

### Fixed query position attention maps for spatial attention probing

The attention flow analysis presented in the preceding section selected query positions at fixed fractional intervals along the flattened token sequence, a strategy that distributes query anchors uniformly across the spatial grid without regard to the structural semantics of the image. This study extends that analysis by adopting a complementary approach in which three semantically motivated fixed query positions are defined explicitly in two-dimensional grid coordinates, namely *Q*[1,5], *Q*[3,3], and *Q*[5,1], corresponding to an upper-right, central, and lower-left location within the 7×7 feature grid respectively. This diagonal arrangement of query anchors is designed to probe the attention distribution from spatially distinct and structurally diverse regions of the feature map simultaneously, and may offer a more interpretable comparison of how attention propagates from different spatial origins across classes with varying object orientations and structural layouts. By fixing the query positions consistently across all ten Fashion MNIST classes, this approach also enables direct cross-class comparison of attention patterns from the same spatial origin, which may reveal class-specific differences in how spatial context is gathered from each region of the feature grid.

The implementation follows a procedure closely related to that of the attention flow analysis, with the key distinction that query positions are specified as explicit two-dimensional grid coordinates rather than fractional indices along the flattened sequence. For each test sample, the averaged attention matrix *A* of shape 49×49 is computed by extracting the query and key projections from the LSA layer, applying softmax along the feature dimension of *Q* and the token dimension of *K*, and averaging the resulting pairwise attention scores across all four attention heads. For each of the three fixed query positions $\left(q_{y},q_{x}\right)$, the corresponding linear token index is computed as $q_{idx}=q_{y}\times W+q_{x}$, and the row $A[q_{idx},:]$ is extracted and reshaped into a 7×7 spatial map representing the attention that the query token at position $\left(q_{y},q_{x}\right)$ distributes across all 49 spatial key tokens. The three resulting maps are stacked and jointly normalized to [0, 1] using the global minimum and maximum across the stack, ensuring that the color encoding remains directly comparable across the three query positions within each sample. Each 7×7 map is subsequently bilinearly upsampled to 28×28 for overlay visualization.

**Fig 8:**
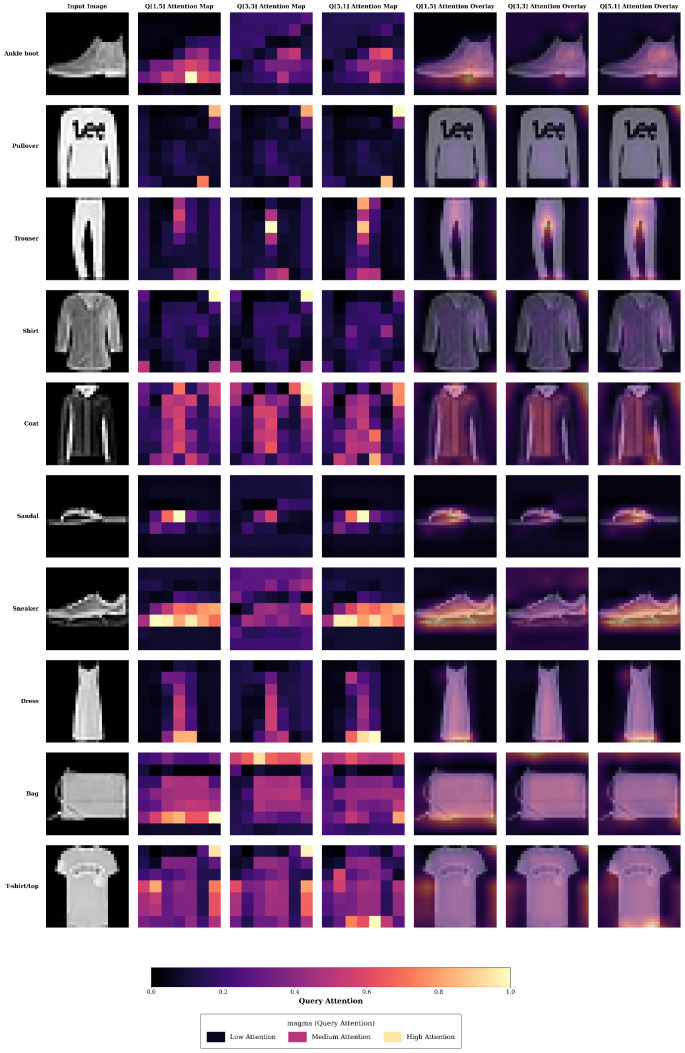
Fixed query position attention maps across all ten Fashion MNIST classes. Each row shows attention maps and overlays for fixed query positions Q[1,5], Q[3,3], and Q[5,1] (magma colormap).

The visualization, presented in Fig 8, is organized as a 10×7 grid where each row corresponds to one correctly classified Fashion MNIST class. Column 1 displays the original grayscale input image. Columns 2–4 display the raw 7×7 attention maps for query positions *Q*[1,5], *Q*[3,3], and *Q*[5,1] respectively, rendered in the *magma* colormap where black and dark purple encode negligible attention and bright yellow and white encode high attention. Columns 5–7 display the corresponding 28×28 bilinearly upsampled overlays of each query’s attention map onto the input image at a transparency of α=0.55, allowing direct spatial alignment between the attention distribution and visible image content.

Examining the attention maps across all ten classes and three query positions reveals patterns that may offer insight into how spatial context is gathered from different regions of the feature grid. A consistent observation across multiple classes is that the attention distribution from each query position tends to exhibit a degree of spatial locality, with higher attention values appearing in regions that are proximate to the query anchor within the 7×7 grid. This locality tendency appears most pronounced for the upper-right query position *Q*[1,5] and the lower-left query position *Q*[5,1], both of which are located near the periphery of the feature grid, and may suggest that peripheral tokens preferentially gather context from their spatial neighborhood rather than attending broadly across the entire feature map. The central query position *Q*[3,3], by contrast, appears to produce somewhat more spatially distributed attention maps for several classes, which may be consistent with the expectation that a centrally located token has access to a broader and more balanced spatial context.

For the Ankle Boot class, the query position *Q*[1,5] produces a concentrated bright region in the upper-right area of the feature map, which when upsampled appears to correspond to the toe and upper body of the boot, suggesting that this peripheral query token attends primarily to the spatially adjacent foreground region of the object. The central query *Q*[3,3] produces a more broadly distributed attention map for this class, with moderate intensity values spread across the central and lower regions of the feature grid, potentially reflecting the midsole and heel area of the boot. The lower-left query *Q*[5,1] produces a notably different pattern, with high attention concentrated in the lower-left region of the map that may correspond to the background or the base of the boot, suggesting that this query position may be encoding boundary information rather than object-interior features. For the Sandal class, all three query positions produce relatively concentrated attention maps that appear to converge on the horizontal mid-region of the image where the sole of the sandal is located, with the brightest regions appearing in the maps for *Q*[1,5] and *Q*[5,1], suggesting that both peripheral query tokens attend strongly to the most structurally salient region of this class regardless of their spatial origin.

The Trouser class presents a particularly informative pattern across the three query positions. The raw attention maps for all three queries reveal vertical band-like structures of elevated attention that align with the central seam region of the trousers, consistent with the seam-anchored attention behavior identified in earlier analyses. Notably, the vertical extent of the high-attention band appears to shift depending on the query position, with *Q*[1,5] producing a band concentrated in the upper portion of the feature grid, *Q*[3,3] producing a band centered in the middle, and *Q*[5,1] producing a band in the lower portion, suggesting that each query token attends to the segment of the seam that is most spatially proximate to its own location. This position-dependent vertical segmentation of attention may be interpreted as a form of implicit structural decomposition, where different spatial tokens collectively encode different portions of the elongated garment structure rather than attending uniformly to the entire object.

For the Pullover and Coat classes, the three query positions produce attention maps with broadly distributed moderate-intensity values that cover much of the garment area, with brighter regions appearing near the query anchor and gradually diminishing intensity toward more distant spatial locations. The Dress class shows a somewhat more structured pattern, with the central query *Q*[3,3] producing a vertically oriented high-attention band along the body of the dress, and the peripheral queries producing more localized attention concentrations near their respective grid positions. The Bag class produces attention maps with relatively broad and uniform distributions across all three query positions, which may reflect the holistic and featureless nature of the bag’s rectangular structure, as suggested by the attention flow and rollout analyses. The T-Shirt/Top and Shirt classes both show elevated attention near the collar and shoulder region for the upper-right query *Q*[1,5], potentially indicating that this region of the feature grid is particularly informative for distinguishing these garment categories.

The fixed query position approach appears to offer a structured and spatially explicit complement to the aggregated attention visualization methods explored in earlier sections. By probing the attention matrix from three geometrically distinct and consistently defined spatial origins, this analysis may reveal positional asymmetries in the attention distribution that aggregated methods would obscure, and the diagonal arrangement of query anchors appears appropriate for capturing attention patterns from upper, central, and lower regions of the feature map simultaneously. The use of the *magma* colormap, distinct from the *viridis* colormap employed in the received attention and per-head analyses, provides a clear visual distinction between the query-specific and aggregate attention visualizations, which assists in differentiating between the two complementary perspectives on spatial attention presented in this study.

### Integrated gradients for pixel-level attribution

The attention-based visualization methods explored in the preceding sections operate exclusively on the internal representations of the LSA module, and as such, their spatial resolution is fundamentally constrained by the 7×7 feature grid produced by the CNN backbone. While bilinear upsampling extends these maps to the original 28×28 resolution for overlay purposes, the underlying attribution signal remains coarse and spatially smoothed. This study complements the attention-based analyses by applying Integrated Gradients (IG), a gradient-based attribution method that operates directly at the pixel level and produces attribution maps at the full input resolution of 28×28, offering a fundamentally different and potentially more spatially precise perspective on which input pixels contribute most to the model’s classification decision.

Integrated Gradients is grounded in axiomatic attribution theory and satisfies two desirable theoretical properties, namely completeness and sensitivity, that many simpler gradient-based methods do not. The method attributes the model’s output to each input feature by accumulating gradients along a straight-line path in the input space from a reference baseline to the actual input. Formally, for an input *x* and a baseline $\bar{x}$, the integrated gradient attribution for input dimension *i* is defined as the integral of the gradient of the model output *F* with respect to input dimension *i*, evaluated along the interpolated path from $\bar{x}$ to *x*, scaled by the input difference $\left(x_{i}-{\bar{x}}_{i}\right)$. In this implementation, the baseline is set to a zero tensor of the same shape as the input, representing a blank black image that carries no class-relevant information, and the path integral is approximated using 50 uniformly spaced interpolation steps, a choice that appears appropriate for balancing computational cost against approximation accuracy. The Captum library is employed for the Integrated Gradients computation, which provides a well-validated and numerically stable implementation of the method. The attribution is computed with respect to the predicted class label for each sample, ensuring that the resulting attribution map reflects the pixel-level evidence that the model relied upon to arrive at its specific classification decision rather than a generic class-agnostic saliency signal.

**Fig 9:**
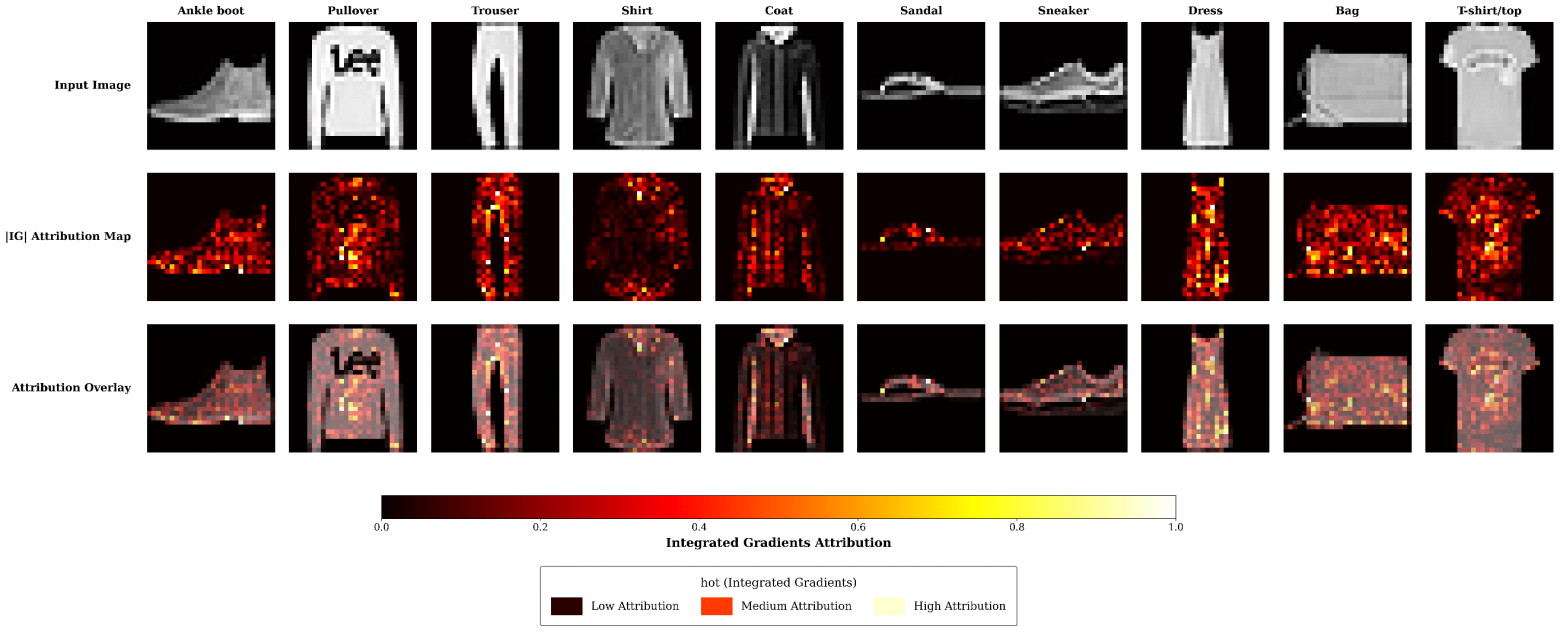
Integrated Gradients attribution maps across all ten Fashion MNIST classes. Each column shows the input image, pixel-wise IG attribution map, and attribution overlay (hot colormap).

The raw Integrated Gradients attributions are signed values that may be positive or negative, where positive values indicate pixels whose intensity increases the model’s confidence in the predicted class and negative values indicate pixels whose intensity suppresses it. For visualization purposes, the absolute value of the attribution map is taken, yielding a magnitude map |*IG*| that reflects the overall importance of each pixel regardless of the direction of its contribution. Each magnitude map is then normalized per sample to [0, 1] by dividing by its maximum value, ensuring visual consistency across classes with varying attribution scales. The visualization, presented in Fig 9, is organized as a 3×10 grid where each column corresponds to one correctly classified Fashion MNIST class. The first row displays the original grayscale input image. The second row displays the normalized |*IG*| magnitude map rendered in the *hot* colormap, where dark red and black encode negligible attribution and bright yellow and white encode the highest pixel-level attribution. The third row displays the |*IG*| magnitude map overlaid on the input image at a transparency of α=0.5, allowing direct spatial correspondence between high-attribution pixels and visible image content to be assessed.

A notable characteristic of the IG attribution maps, relative to the attention-based visualizations, is that the resulting maps appear considerably more spatially fine-grained and pixel-precise, exhibiting attribution patterns that closely follow the visible edges, textures, and structural boundaries of the objects in the input images. This increased spatial resolution may be attributed to the fact that Integrated Gradients operates directly on the input pixel space rather than on the coarse 7×7 feature map, and the gradient signal propagates through the full model architecture including both the CNN backbone and the LSA module, potentially capturing the combined contribution of all model components to the final classification decision. The attribution maps also exhibit a characteristically granular or pixelated texture at the local level, which may reflect the sensitivity of gradient-based methods to fine-grained pixel variations and is a commonly observed property of IG attributions in image classification tasks.

For the Ankle Boot class, the |*IG*| magnitude map reveals high attribution concentrated along the visible edges and textural details of the boot, particularly around the toe cap, sole boundary, and the upper body of the boot where the leather texture transitions to the background. The bright yellow and white pixels in the second row appear to correspond closely to the structural contours of the boot that are most visually distinctive and discriminative. For the Trouser class, the IG map produces a striking vertical band of high attribution along the central seam and inner leg region of the trousers, consistent with the seam-anchored attention patterns identified in the LSA-based analyses, and suggesting that both the attention mechanism and the gradient-based attribution method converge on the same structurally distinctive feature for this class. This cross-method consistency may be interpreted as a form of convergent evidence supporting the interpretability of the model’s learned representations.

For the Sandal and Sneaker classes, the Integrated Gradients maps reveal high attribution concentrated along the sole and midsole regions of the footwear, with bright pixels closely following the horizontal structural boundary between the shoe and the background. The Sandal class in particular produces a notably sparse attribution map with high-intensity pixels confined to a narrow horizontal band corresponding to the sole, which may reflect the relatively simple and compact structural profile of this class. The Sneaker class produces a somewhat broader attribution pattern that extends across the midsole and upper body of the shoe, consistent with the more complex and spatially extended structure of this category. For the Coat class, the IG map reveals high attribution distributed along the vertical seams and collar region of the coat, with bright pixels appearing at the boundaries between the coat body and the darker background, suggesting that edge and boundary information may be particularly important for distinguishing this class from visually similar garment categories such as the Pullover and Shirt.

The Pullover class presents an interesting pattern in which high attribution appears both along the garment boundaries and within the interior of the garment, particularly in the region corresponding to the printed logo visible in the input image. This observation may suggest that the model attends to both structural shape features and interior texture features when classifying the Pullover, and the IG method appears capable of capturing this dual reliance in a way that the coarser attention maps may not fully resolve. The Dress class shows high attribution concentrated along the shoulder straps and the central vertical axis of the dress body, consistent with the vertically oriented attention patterns observed in earlier analyses. The Bag class produces attribution maps with relatively broadly distributed high-intensity pixels across the rectangular body of the bag, including along the edges and handle region, which may reflect the holistic boundary encoding strategy suggested by the attention-based analyses. The T-Shirt/Top class shows elevated attribution around the collar and sleeve boundaries, with the overlay map revealing that the high-attribution pixels align closely with the structural contours of the garment.

The Integrated Gradients method appears to offer a potentially valuable complement to the attention-based XAI techniques explored in this study, providing pixel-level attribution maps that are grounded in axiomatic theory and operate at the full input resolution rather than the coarse spatial resolution of the attention feature grid. The degree of spatial consistency observed between the IG attribution maps and the attention-based saliency maps across multiple classes may suggest that the model’s learned representations are interpretable and spatially coherent across different XAI methodologies, which could be interpreted as a positive indicator of the model’s overall transparency. One limitation is that the granular and pixelated texture of Integrated Gradients maps may make them more difficult to interpret visually compared to the smoother attention overlays, and the sensitivity of gradient-based methods to local pixel variations may introduce noise that does not necessarily reflect semantically meaningful attribution.

### LIME-based superpixel attribution for model-agnostic explainability

All XAI techniques explored in the preceding sections are intrinsic to the TinyCNN-LSA architecture in the sense that they either directly interrogate the model’s internal attention weights or compute gradients with respect to the model’s parameters. While such approaches may offer mechanistically faithful explanations, they are inherently model-specific and cannot be straightforwardly applied to architectures that do not expose differentiable internal representations. This study complements the intrinsic analyses by applying Local Interpretable Model-Agnostic Explanations (LIME), a post-hoc surrogate-based XAI method that treats the model as a black box and constructs locally faithful linear approximations of its decision boundary in the neighborhood of each input sample. The model-agnostic nature of LIME makes it a potentially valuable cross-validation tool, as the spatial attribution patterns it produces are derived entirely from the model’s input-output behavior rather than from its internal representations, and any consistency between LIME attributions and the attention or gradient-based maps explored previously may be interpreted as convergent evidence supporting the interpretability of the model’s learned spatial reasoning.

LIME operates by generating a set of perturbed versions of the input image, obtaining the model’s predictions for each perturbed sample, and fitting a sparse linear model in the space of interpretable superpixel features that locally approximates the relationship between superpixel presence and model output confidence. In this implementation, the input image is first converted to a three-channel RGB representation by replicating the single grayscale channel across all three color dimensions, as the LIME image explainer operates on RGB inputs. The image is then segmented into superpixels using the Quickshift algorithm with a kernel size of 1, a maximum distance of 3, and a ratio of 0.2, parameter choices that appear appropriate for producing fine-grained superpixel segments that respect the local structure of the 28×28 Fashion MNIST images without over-segmenting the relatively low-resolution input. A total of 1000 perturbed samples are generated per explanation, a count that appears sufficient for producing stable linear approximations while remaining computationally tractable. The prediction function passed to the LIME explainer extracts the first channel of the RGB input, converts it to a single-channel tensor, and passes it through the TinyCNN-LSA model to obtain class probability outputs via softmax, ensuring that the LIME surrogate is trained on the same probability distribution that the model produces during standard inference.

**Fig 10:**
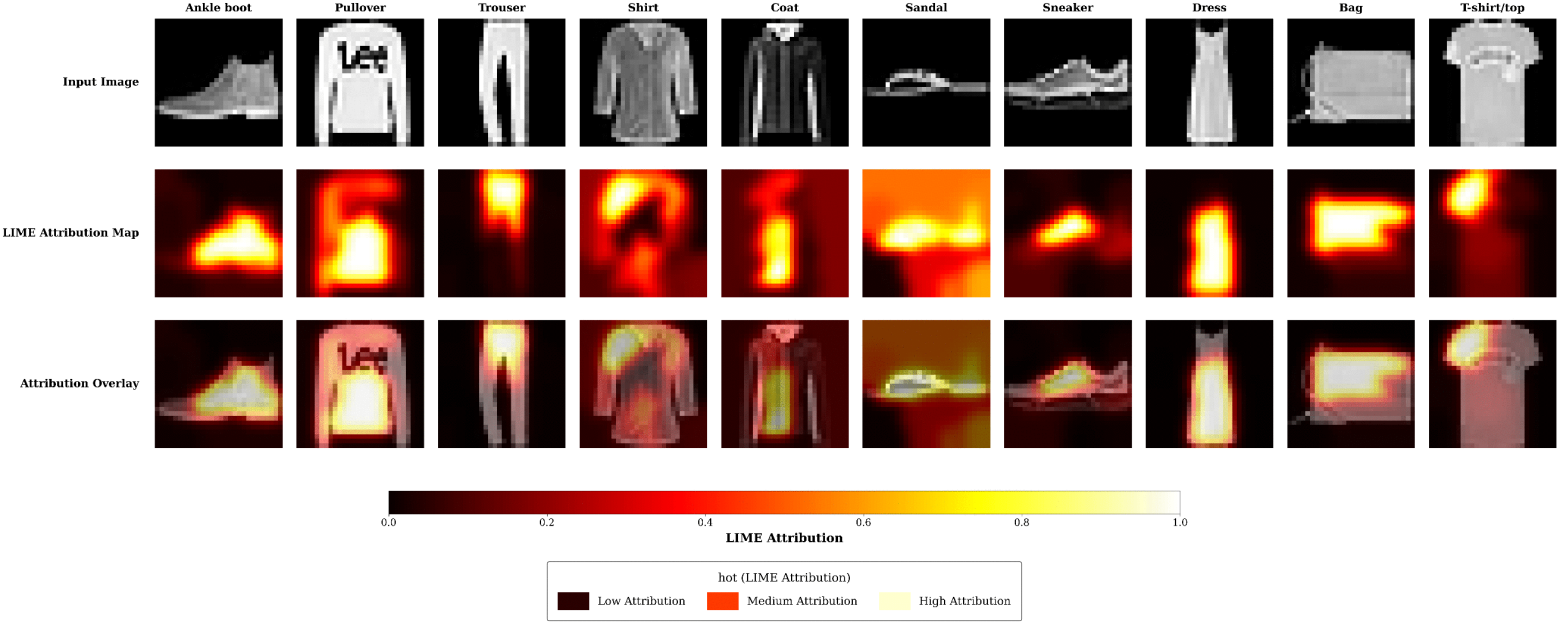
LIME superpixel attribution maps across all ten Fashion MNIST classes. Each column shows the input image, LIME superpixel attribution map, and attribution overlay (hot colormap).

The LIME explanation for each sample yields a set of superpixel weights associated with the predicted class, where positive weights indicate superpixels whose presence increases the model’s confidence in the predicted class and negative weights indicate superpixels whose presence suppresses it. A raw attribution heatmap is constructed by assigning each pixel the weight of the superpixel to which it belongs. The absolute value of this heatmap is then taken to produce a magnitude map that reflects the overall importance of each superpixel region regardless of the direction of its contribution, consistent with the absolute attribution approach adopted for the Integrated Gradients analysis. A Gaussian smoothing filter with a standard deviation of σ=1.5 is subsequently applied to the magnitude map, producing a spatially continuous saliency surface that reduces the blocky superpixel boundaries and yields a more visually coherent attribution map. Each smoothed map is normalized per sample to [0, 1] by dividing by its maximum value, ensuring visual consistency across classes. The visualization, presented in Fig 10, is organized as a 3×10 grid where each column corresponds to one correctly classified Fashion MNIST class. The first row displays the original grayscale input. The second row displays the smoothed normalized |*LIME*| magnitude map in the *hot* colormap, where dark red and black encode negligible attribution and bright yellow and white encode the highest superpixel importance. The third row displays the magnitude map overlaid on the input image at a transparency of α=0.5.

A visually striking characteristic of the LIME attribution maps, relative to the Integrated Gradients and attention-based visualizations, is that the resulting saliency regions appear considerably smoother and more spatially coherent, forming large contiguous blobs of high attribution that correspond to semantically meaningful regions of the object rather than fine-grained pixel-level patterns. This smoothness arises from the superpixel representation underlying LIME, which groups spatially adjacent and visually similar pixels into unified segments and assigns a single attribution weight to each segment, producing region-level rather than pixel-level explanations. The subsequent Gaussian smoothing further enhances this spatial coherence, and the resulting maps appear more immediately interpretable to human observers than the granular IG maps, though at the cost of pixel-level spatial precision.

For the Ankle Boot class, the LIME attribution map produces a large bright region concentrated on the body and sole of the boot, with the highest attribution values appearing in the central foreground area of the object and gradually diminishing toward the background. The overlay map confirms that this high-attribution region aligns closely with the visible structural extent of the boot, suggesting that the LIME surrogate identifies the overall boot silhouette as the most important input region for this classification. For the Trouser class, the LIME map produces a narrow vertical band of high attribution along the central axis of the trousers, consistent with the seam-anchored patterns identified by the attention-based analyses and the Integrated Gradients method, and representing a notable instance of cross-method convergence for this class. The vertical extent and spatial position of the high-attribution band in the LIME map appears broadly consistent with the seam region identified by the LSA attention maps, suggesting that this structural feature may be genuinely discriminative for the Trouser class across multiple attribution methodologies.

For the footwear classes, the LIME maps reveal attribution patterns that appear broadly consistent with those observed in the attention and gradient-based analyses. The Sandal class produces a prominent bright region concentrated along the horizontal sole structure of the sandal, with a smooth gradient that extends slightly above and below the sole boundary. The Sneaker class similarly shows high attribution concentrated along the midsole and lower body of the shoe, though the LIME map for this class appears to extend the high-attribution region somewhat more broadly than the corresponding IG map, which may reflect the coarser spatial resolution of the superpixel representation relative to the pixel-level IG attribution. For the Coat class, the LIME map produces a distinctive pattern with high attribution concentrated in the upper-central region of the garment, corresponding approximately to the collar and upper torso area, with a secondary region of elevated attribution along the lower body of the coat. This two-region attribution pattern may suggest that the LIME surrogate identifies both the collar structure and the overall garment silhouette as important features for distinguishing the Coat class from visually similar garment categories.

The Pullover class produces a LIME attribution map with a large bright region covering the upper-left portion of the garment, which when examined alongside the overlay map appears to correspond to the sleeve and shoulder area on one side of the pullover, as well as the region containing the printed logo. This pattern may suggest that the model relies on a combination of structural shape features and interior texture features for classifying the Pullover, consistent with the observation made in the IG analysis. The Dress class shows a vertically oriented high-attribution region along the central body of the dress, with the brightest pixels concentrated in the upper torso and strap area, consistent with the vertically structured attention patterns identified in earlier analyses. The Bag class produces a notably broad attribution map that covers much of the rectangular body of the bag, with high attribution extending to the edges and corners of the object, potentially reflecting the holistic boundary-encoding strategy suggested by multiple preceding analyses. The T-Shirt/Top class shows high attribution concentrated in the collar and shoulder region on one side of the garment, with the overlay map revealing that the bright region aligns with the visible neckline and sleeve structure.

The LIME-based attribution analysis appears to offer a potentially valuable and methodologically distinct complement to the intrinsic XAI techniques explored in this study. The model-agnostic nature of LIME means that its attribution maps are derived solely from the model’s input-output behavior, and the degree of spatial consistency observed between the LIME maps and the attention-based and gradient-based analyses across multiple classes may be interpreted as convergent evidence suggesting that the model’s spatial reasoning is coherent and interpretable across different explanation paradigms. One consideration worth acknowledging is that LIME explanations are inherently stochastic, as the perturbed samples are generated randomly and the linear surrogate is fitted locally, meaning that repeated runs may produce somewhat different attribution maps for the same input. The use of a fixed random seed in this implementation appears appropriate for ensuring reproducibility of the reported results. Additionally, the choice of Quickshift segmentation parameters may influence the granularity of the superpixel representation and consequently the spatial resolution of the attribution maps, and different parameter choices could potentially yield different attribution patterns, a sensitivity that may be worth investigating in future work.

### Gradient-based SHAP for pixel-level feature importance estimation

The LIME-based analysis explored in the preceding section offers a model-agnostic perspective on spatial attribution through local surrogate approximation, but its reliance on superpixel perturbations and a stochastic sampling procedure introduces a degree of approximation that may limit the precision of the resulting attribution maps. This study applies a complementary gradient-based attribution approach through SHAP (SHapley Additive exPlanations) using the GradientExplainer variant, which combines the theoretical foundations of Shapley value decomposition with the computational efficiency of gradient-based attribution to produce pixel-level feature importance estimates that are grounded in cooperative game theory. The Shapley value framework gives a set of axiomatic properties including efficiency, symmetry, dummy, and additivity, which collectively ensure that the attribution assigned to each input feature represents a theoretically principled measure of that feature’s marginal contribution to the model’s output relative to a reference distribution of background samples. This theoretical grounding may make SHAP a particularly appropriate complement to the heuristic perturbation-based LIME approach and the path-integral-based Integrated Gradients method explored previously.

The GradientExplainer variant of SHAP approximates Shapley values by combining backpropagated gradients with expectations computed over a background dataset, drawing on the connection between expected gradients and Shapley values established in the SHAP theoretical framework. In this implementation, a background dataset of 100 training samples is constructed by drawing batches from the training data loader until the required count is reached. This background dataset serves as the reference distribution against which the marginal contribution of each pixel in the test sample is measured, and the choice of 100 background samples appears appropriate for providing a stable estimate of the expected gradient while remaining computationally feasible. The SHAP GradientExplainer is initialized with the trained TinyCNN-LSA model and the background dataset, and for each test sample the SHAP values are computed with respect to the predicted class label. The resulting SHAP value tensor is extracted and processed through a dimension-handling routine that accommodates the various output shapes that the SHAP library may produce depending on the model architecture and input configuration, ensuring robust extraction of the per-pixel attribution map for the predicted class. The absolute value of the SHAP attribution map is taken to produce a magnitude map |*SHAP*| that reflects the overall importance of each pixel regardless of the sign of its contribution, and each map is normalized per sample to [0, 1] by dividing by its maximum value to ensure visual consistency across classes.

A visually prominent characteristic of the SHAP attribution maps, relative to both the LIME and Integrated Gradients analyses, is that the resulting maps exhibit a notably granular and spatially distributed attribution pattern, with high-importance pixels appearing in a somewhat scattered arrangement across the image rather than forming the large contiguous blobs observed in the LIME maps or the edge-following patterns observed in the IG maps. This distributed and granular texture may be attributed to the nature of the GradientExplainer approximation, which computes expected gradients over a background distribution and may produce attribution maps that reflect the sensitivity of the model’s output to local pixel variations averaged across multiple reference samples rather than the deterministic gradient at a single baseline point. Despite this granularity, the SHAP maps appear to retain meaningful spatial structure, with regions of elevated attribution broadly corresponding to the foreground object regions rather than the background, and certain class-specific structural features appearing consistently highlighted across the attribution maps.

**Fig 11:**
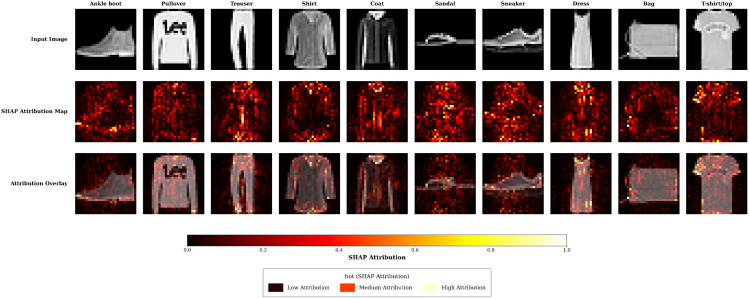
SHAP GradientExplainer attribution maps across all ten Fashion MNIST classes. Each column shows the input image, pixel-wise SHAP attribution map, and attribution overlay (hot colormap).

Fig 11 shows the SHAP visualization, organized as a 3×10 grid where each column corresponds to one correctly classified Fashion MNIST class. The first row displays the original grayscale input image. The second row displays the normalized |*SHAP*| magnitude map rendered in the *hot* colormap, where dark red and black encode negligible feature importance and bright yellow and white encode the highest pixel-level SHAP attribution. The third row displays the magnitude map overlaid on the input image at a transparency of α=0.5, allowing direct spatial correspondence between high-attribution pixels and visible image content to be assessed.

For the Ankle Boot class, the SHAP magnitude map reveals broadly distributed attribution across the body of the boot, with moderately elevated values appearing throughout the foreground region of the object and scattered bright pixels appearing at various locations along the boot’s structural boundaries. The overlay map confirms that the high-attribution region aligns with the visible extent of the boot, though the attribution appears less sharply localized than the corresponding IG or LIME maps. For the Trouser class, the SHAP map produces a pattern with relatively elevated attribution along the central vertical axis of the trousers, consistent with the seam-anchored attribution behavior identified across multiple preceding analyses, though the SHAP attribution for this class appears somewhat more diffuse than the sharply defined vertical bands observed in the IG and attention-based maps. The persistence of the seam-region attribution across SHAP, IG, LIME, and the attention-based methods may be interpreted as strong convergent evidence that the central seam of the Trouser class constitutes a genuinely discriminative structural feature that the model has learned to rely upon across its full computational pathway.

For the footwear classes, the SHAP maps reveal attribution patterns that appear broadly consistent with those observed in the preceding analyses, though with greater spatial diffuseness. The Sandal class produces elevated attribution in the horizontal mid-region of the image corresponding to the sole structure, with scattered bright pixels appearing along the boundary between the sandal and the background. The Sneaker class similarly shows elevated attribution in the midsole and lower body region of the shoe, with the overlay map revealing that the high-attribution pixels align with the visible structural extent of the sneaker. The Coat class produces a SHAP map with elevated attribution distributed across the torso and collar region of the coat, with a notably bright pixel appearing in the upper-central area of the feature map that may correspond to the collar or lapel structure. The Shirt class shows a similar pattern with elevated attribution in the upper torso and collar area, and the overlay map reveals that the high-attribution region covers much of the visible garment area.

The Pullover class produces a SHAP attribution map with elevated values distributed across the garment body, with moderately bright regions appearing in the area corresponding to the printed logo as well as along the shoulder and sleeve boundaries. This pattern appears broadly consistent with the IG and LIME findings for this class, suggesting that the model may rely on both interior texture and structural boundary features for classifying the Pullover. The Dress class shows elevated SHAP attribution along the central vertical axis and upper body of the dress, consistent with the vertically oriented attribution patterns identified across multiple preceding analyses. The Bag class produces a SHAP map with relatively broadly distributed attribution across the rectangular body of the bag, with elevated values appearing along the edges and corners of the object, consistent with the boundary-encoding attribution pattern suggested by the LIME and attention-based analyses. The T-Shirt/Top class shows scattered elevated attribution across the collar and shoulder region, with the overlay map revealing that the high-attribution pixels broadly correspond to the visible structural features of the garment.

The SHAP GradientExplainer analysis appears to offer a theoretically grounded and computationally appropriate complement to the other XAI methods explored in this study. The axiomatic properties of the Shapley value framework may provide a stronger theoretical justification for the resulting attributions than heuristic perturbation or gradient-based methods, and the use of a background dataset to define the reference distribution appears more principled than the fixed zero-baseline employed in the Integrated Gradients analysis. The granular and spatially distributed nature of the SHAP maps relative to the LIME and IG maps may make them somewhat less immediately interpretable to human observers, though the overall spatial correspondence between high-attribution regions and foreground object structures appears to remain consistent across all three pixel-level attribution methods explored in this study.

#### Quantitative analysis of attention distribution via entropy, sparsity, and maximum attention statistics.

The qualitative attention visualizations explored in the preceding sections offer spatially intuitive representations of where the LSA module directs its attention across different input classes, but they do not provide a systematic quantitative characterization of the statistical properties of the attention distribution across the broader test set. This study addresses this gap by computing three complementary scalar statistics over the averaged attention matrix for 200 test samples, namely attention entropy, attention sparsity, and maximum attention value, and analyzing their distributions and pairwise relationships. These statistics collectively characterize whether the attention mechanism of the TinyCNN-LSA model operates in a concentrated or diffuse regime, and offer quantitative support for the qualitative observations made in the visualization-based analyses.

#### Attention matrix preparation.

For each test sample, the raw attention matrix is first extracted from the LSA module. The CNN backbone produces a feature map of shape B×C×H×W, which is reshaped into a token sequence of shape B×N×C where N=H×W=7×7=49. The query and key projections are obtained from the shared linear projection and split into per-head representations of dimensionality *d* = *C* / *h* where *h* = 4 denotes the number of attention heads. Softmax is applied along the feature dimension of the query matrix and along the token dimension of the key matrix, following the linear attention formulation. The per-head pairwise attention scores are then computed as:


$$\begin{array}{c} A^{\left(l\right)}=Q^{(l)⊤}K^{\left(l\right)},\;l=1,2,3,4 \end{array}$$ (30)

where $A^{\left(l\right)}\in {\mathbb{R}}^{N\times N}$ denotes the attention matrix for head *l*, and $Q^{\left(l\right)},K^{\left(l\right)}\in {\mathbb{R}}^{N\times d}$ denote the softmax-normalized query and key matrices for that head. The four per-head attention matrices are averaged to produce the samp*l*e-level averaged attention matrix:


$$\begin{array}{c} A=\frac{1}{h}\sum_{l=1}^{h}A^{\left(l\right)},\;A\in {\mathbb{R}}^{N\times N} \end{array}$$ (31)

Each row of *A* is subsequently normalized to sum to unity by dividing by its row sum, yielding the row-stochastic normalized attention matrix:


$$\begin{array}{c} \hat{A}[i,j]=\frac{A[i,j]}{\sum_{k=1}^{N}A[i,k]+\epsilon },\;\forall \;i,j\in \{1,\ldots ,N\} \end{array}$$ (32)

where $\epsilon =10^{-12}$ is a small constant added for numerical stability. Each entry $\hat{A}[i,j]$ can be interpreted as the normalized attention weight that spatial token *i* assigns to spatial token *j*, and each row of $\hat{A}$ constitutes a valid probability distribution over the *N* = 49 spatial key tokens.

#### Attention entropy.

The attention entropy quantifies the degree of uniformity or concentration in the attention distribution. For a given sample, the per-row Shannon entropy is first computed for each query token *i* as:


$$\begin{array}{c} H_{i}=-\sum_{j=1}^{N}\hat{A}[i,j]\log\left(\hat{A}[i,j]+\epsilon \right),\;i=1,\ldots ,N \end{array}$$ (33)

The sample-level attention entropy is then obtained by averaging the per-row entropies across all *N* query tokens:


$$\begin{array}{c} H=\frac{1}{N}\sum_{i=1}^{N}H_{i}=-\frac{1}{N}\sum_{i=1}^{N}\sum_{j=1}^{N}\hat{A}[i,j]\log\left(\hat{A}[i,j]+\epsilon \right) \end{array}$$ (34)

The theoretical bounds for this entropy measure are established by the properties of the Shannon entropy function. For a theoretically uniform attention distribution where $\hat{A}[i,j]=1/N$ for all *i*, *j*, the entropy achieves its maximum value:


$$\begin{array}{c} H_{\max}=\log(N)=\log(49)\approx 3.892\text{ nats} \end{array}$$ (35)

**Fig 12:**
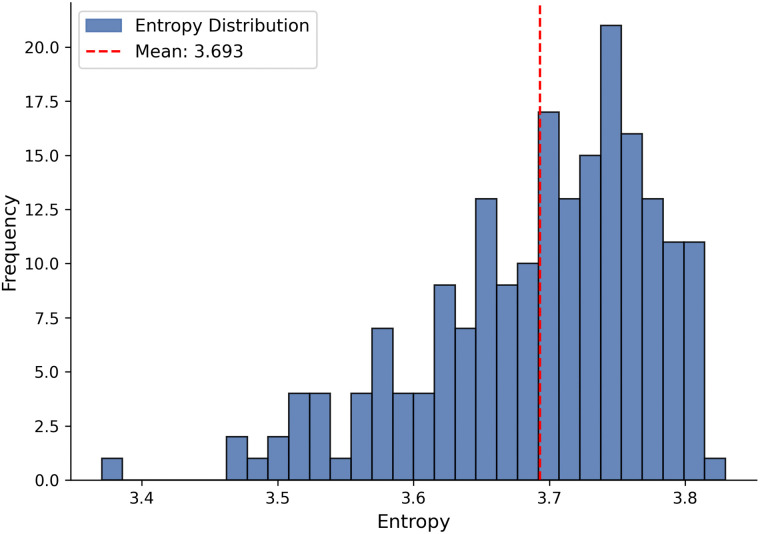
Distribution of attention entropy across 200 test samples. Histogram of LSA attention entropy values over 200 test samples, with the mean marked by a red dashed line (3.693).

For a theoretically concentrated one-hot distribution where a single key token receives all attention from each query, the entropy achieves its minimum value of $H_{\min}=0$ nats. The observed mean entropy of $\bar{H}=3.6930\pm 0.0837$ nats corresponds to:


$$\begin{array}{c} \frac{\bar{H}}{H_{\max}}=\frac{3.6930}{3.892}\approx 0.9489 \end{array}$$ (36)

indicating that the observed attention entropy is approximately 94.9% of the theoretical maximum, which suggests that the attention distribution of the TinyCNN-LSA model is broadly diffuse across the majority of test samples. As shown in Fig 12, the entropy histogram spans approximately the range [3.35, 3.85] nats with the bulk of the mass concentrated between 3.65 and 3.85 nats, confirming this broadly diffuse regime. The standard deviation of $\sigma_{H}=0.0837$ relative to the mean further suggests that this diffuse attentional regime is maintained consistently across different input samples rather than fluctuating substantially between inputs.

#### Attention sparsity.

The attention sparsity metric quantifies the degree to which each query token concentrates its attention on a single dominant key token, and is defined as the mean of the per-row maximum attention weights across all query tokens:


$$\begin{array}{c} S=\frac{1}{N}\sum_{i=1}^{N}\max_{j\in \{1,\ldots ,N\}}\hat{A}[i,j] \end{array}$$ (37)

The theoretical bounds for this statistic are derived from the properties of the row-stochastic matrix $\hat{A}$. For a theoretically uniform distribution, the maximum per-row attention weight equals 1/*N*, yielding:


$$\begin{array}{c} S_{\min}=\frac{1}{N}=\frac{1}{49}\approx 0.0204 \end{array}$$ (38)

For a theoretically concentrated one-hot distribution, the maximum per-row weight equals 1.0, yielding $S_{\max}=1.0$. The observed mean sparsity of $\bar{S}=0.0814\pm 0.0336$ may be compared to the uniform baseline through the normalized excess sparsity:


$$\begin{array}{c} \Delta S=\bar{S}-S_{\min}=0.0814-0.0204=0.0610 \end{array}$$ (39)

**Fig 13:**
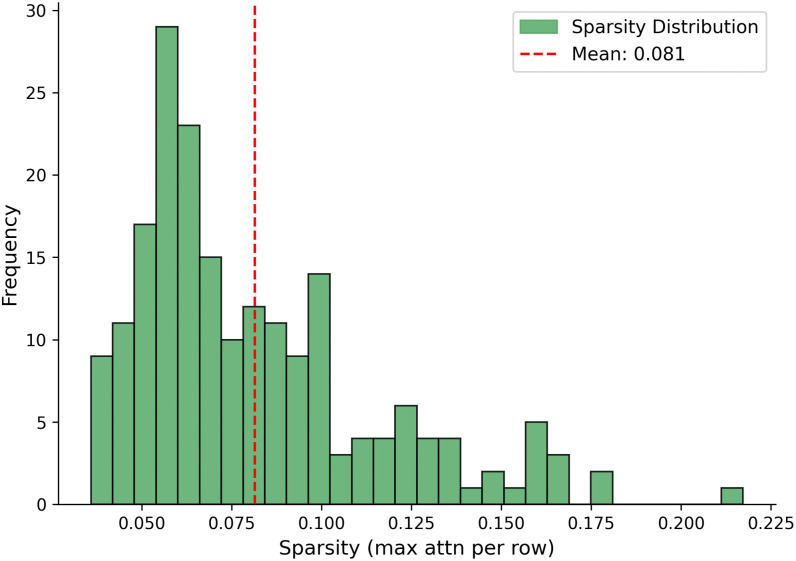
Distribution of attention sparsity across 200 test samples. Histogram of LSA attention sparsity values (max attention per row) over 200 test samples, with the mean marked by a red dashed line (0.081).

This excess of approximately 0.0610 above the uniform baseline suggests that the attention mechanism exhibits a meaningful degree of preferential concentration toward dominant key tokens beyond what would be expected under a fully diffuse uniform distribution. Furthermore, the ratio of the observed mean sparsity to the uniform baseline:


$$\begin{array}{c} \frac{\bar{S}}{S_{\min}}=\frac{0.0814}{0.0204}\approx 3.99 \end{array}$$ (40)

indicates that the average maximum per-row attention weight is approximately 3.99 times larger than the uniform expectation, suggesting a degree of selective concentration that, while moderate, may be considered non-trivial. The right-skewed distribution of sparsity values observed in Fig 13, with the majority of samples concentrated in the range [0.04, 0.10] and a tail extending to values approaching 0.225, is consistent with the interpretation that most samples exhibit moderate concentration while a subset of samples, potentially corresponding to compact and structurally distinctive objects, elicits notably higher attentional selectivity.

#### Maximum attention value.

The maximum attention value captures the peak attention intensity across all token pairs within a given sample, and is defined as the global maximum of the normalized attention matrix:


$$\begin{array}{c} M=\max_{i\in \{1,\ldots ,N\},\;j\in \{1,\ldots ,N\}}\hat{A}[i,j] \end{array}$$ (41)

Unlike the sparsity statistic which averages the per-row maxima, *M* reflects the single most concentrated attention relationship present anywhere in the attention matrix for a given sample, and may be interpreted as an upper bound on the degree to which any individual spatial token pair dominates the attention distribution. The observed mean maximum attention value of $\bar{M}=0.1134\pm 0.0511$ may be compared to the uniform baseline through the ratio:


$$\begin{array}{c} \frac{\bar{M}}{S_{\min}}=\frac{0.1134}{0.0204}\approx 5.56 \end{array}$$ (42)

indicating that the average peak attention weight is approximately 5.56 times larger than the uniform expectation of 1/49, suggesting that the model does identify and preferentially attend to dominant spatial position pairs to a meaningful degree even within the broadly diffuse attentional regime suggested by the entropy statistics. The standard deviation of $\sigma_{M}=0.0511$ reflects a moderate degree of variability in the peak attention intensity across samples, consistent with the observation that different input classes elicit different degrees of attentional concentration as evidenced by the qualitative visualization analyses.

#### Entropy-sparsity relationship and joint analysis.

**Fig 14:**
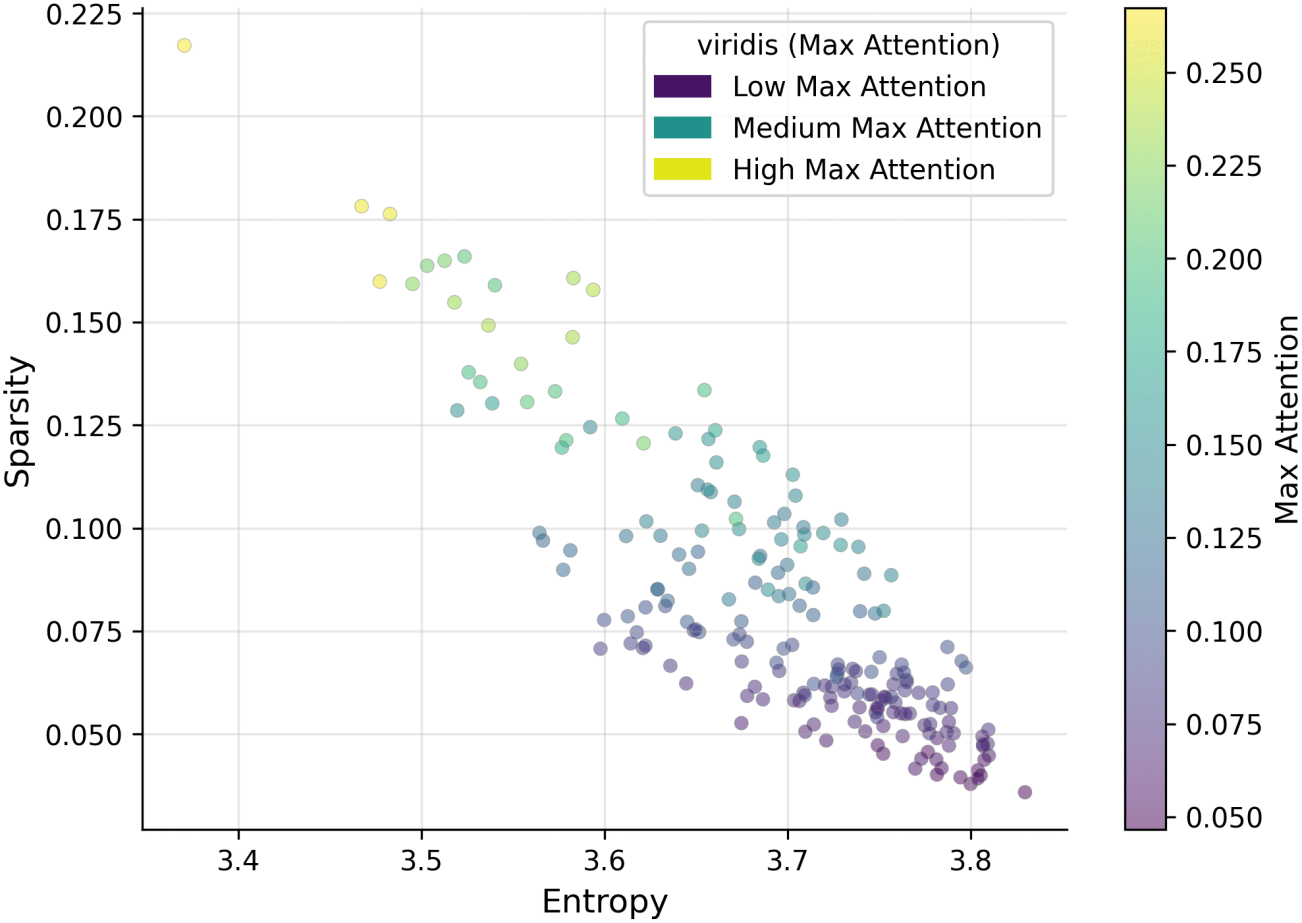
Scatter plot of attention entropy versus sparsity, colored by maximum attention value. Each point represents one test sample, plotting attention entropy against sparsity, with color indicating the maximum attention value (viridis colormap).

The scatter plot presented in Fig 14 reveals a visually prominent negative relationship between entropy *H* and sparsity *S* across the 200 test samples. This relationship is theoretically grounded in the properties of the Shannon entropy and the maximum operator. For a row-stochastic distribution $\hat{A}[i,:]$, a higher maximum value $\max_{j}\hat{A}[i,j]$ necessarily implies greater concentration of probability mass on fewer tokens, which simultaneously reduces the entropy of that distribution. Conversely, a lower maximum value implies more uniform distribution of probability mass, which simultaneously increases the entropy. This inverse relationship may be expressed qualitatively as:


$$\begin{array}{c} H↑⟹S↓,\;H↓⟹S↑ \end{array}$$ (43)

The scatter plot further encodes the maximum attention value *M* through the *viridis* colormap, where deep purple indicates low *M* values in the approximate range [0.05, 0.08] and bright yellow indicates high *M* values approaching 0.275. A consistent three-way pattern emerges in which samples with low entropy and high sparsity, appearing in the upper-left region of the scatter plot, exhibit bright yellow or green coloring indicating high *M*, while samples with high entropy and low sparsity, appearing in the lower-right region, exhibit deep blue and purple coloring indicating low *M*. This three-way relationship between *H*, *S*, and *M* is internally consistent and may be summarized as:


$$\begin{array}{c} H↓,\;S↑,\;M↑\;⟺\;\text{concentrated attentional regime} \end{array}$$ (44)

$$\begin{array}{c} H↑,\;S↓,\;M↓\;⟺\;\text{diffuse attentional regime} \end{array}$$ (45)

The modal cluster of samples with *H* > 3.70 and *S* < 0.08, constituting the largest and most densely populated region of the scatter plot, corresponds to the diffuse attentional regime defined in Eq 45, while the smaller cluster of samples with *H* < 3.60 and *S* > 0.12 in the upper-left region corresponds to the concentrated attentional regime defined in Eq 44. These two clusters may tentatively correspond to structurally complex garment classes that elicit diffuse global attention and structurally compact object classes such as sandals and ankle boots that elicit more localized and concentrated attention, consistent with the qualitative observations from the attention visualization analyses.

Taken together, the three quantitative statistics and their joint distribution provide a coherent and mathematically grounded characterization of the attentional behavior of the TinyCNN-LSA model. The mean entropy of 3.6930 nats at approximately 94.9% of the theoretical maximum, the mean sparsity of 0.0814 at approximately 3.99 times the uniform baseline, and the mean maximum attention of 0.1134 at approximately 5.56 times the uniform baseline collectively suggest that the model operates in a broadly diffuse but selectively concentrated attentional regime, where attention is distributed across many spatial tokens while still exhibiting meaningful preferential concentration toward structurally relevant positions. This quantitative characterization is broadly consistent with the qualitative patterns observed across the attention visualization analyses presented in the preceding sections, and offers numerical support for the interpretation that the LSA module has learned a spatially distributed yet selectively focused attention strategy appropriate for the Fashion image recognition task.

### Prediction confidence and uncertainty analysis

To complement the attention-based interpretability analyses presented in the preceding sections, this study conducts a systematic quantitative examination of the model’s predictive confidence and uncertainty characteristics across the test set. While attention statistics characterize the internal representational behaviour of the LSA module, confidence and uncertainty analysis characterizes the model’s output-level behaviour, specifically the degree to which the softmax probability distribution over the ten Fashion MNIST classes is peaked or diffuse, and how this distributional behaviour relates to prediction correctness. This analysis is organized around four complementary visualizations: a confidence distribution histogram stratified by prediction correctness, a per-class average confidence bar chart, a top confusion pair chart, and a confidence versus entropy scatter plot.

#### Softmax probability and confidence extraction.

For each test sample with input image **x**, the TinyCNN-LSA model produces a raw logit vector $𝐳\in {\mathbb{R}}^{C}$ where *C* = 10 denotes the number of classes. The logit vector is converted to a probability distribution via the softmax function:


$$\begin{array}{c} p_{c}=\frac{\exp(z_{c})}{\sum_{j=1}^{C}\exp(z_{j})},\;c=1,\ldots ,C \end{array}$$ (46)

yielding a probability vector $𝐩=(p_{1},p_{2},\ldots ,p_{C}{)}^{⊤}\in {\mathbb{R}}^{C}$ satisfying $p_{c}\geq 0$ for all *c* and $\sum_{c=1}^{C}p_{c}=1$. Each entry $p_{c}$ may be interpreted as the model’s estimated posterior probability that the input **x** belongs to class *c*. The predicted class label is obtained by taking the argmax of the probability vector:


$$\begin{array}{c} \hat{y}=\text{arg}\max_{c\in \{1,\ldots ,C\}}p_{c} \end{array}$$ (47)

The prediction confidence for a given sample is defined as the maximum class probability, that is, the probability assigned to the predicted class:


$$\begin{array}{c} \hat{p}=\max_{c\in \{1,\ldots ,C\}}p_{c}=p_{\hat{y}} \end{array}$$ (48)

A value of $\hat{p}$ close to 1.0 indicates that the model assigns nearly all probability mass to a single class, reflecting a highly confident and decisive prediction. A value of $\hat{p}$ close to 1/*C* = 0.1 for the ten-class case indicates that the probability mass is distributed nearly uniformly across all classes, reflecting a highly uncertain and indecisive prediction. The prediction entropy is defined as the Shannon entropy of the softmax probability vector:


$$\begin{array}{c} H(𝐩)=-\sum_{c=1}^{C}p_{c}\ln(p_{c}+\epsilon ) \end{array}$$ (49)

where $\epsilon =10^{-10}$ is a small constant added for numerical stability. The entropy H(𝐩) provides a complementary measure of predictive uncertainty: higher entropy values indicate a more diffuse and uncertain probability distribution, while lower entropy values indicate a more concentrated and confident distribution. The theoretical bounds for the prediction entropy are:


$$\begin{array}{c} H_{\min}=0\text{ nats}\;\text{(one-hot distribution, }\hat{p}=1.0\text{)} \end{array}$$ (50)

$$\begin{array}{c} H_{\max}=\ln(C)=\ln(10)\approx 2.303\text{ nats}\;\text{(uniform distribution, }\hat{p}=0.1\text{)} \end{array}$$ (51)

The functional relationship between $\hat{p}$ and H(𝐩) is monotonically decreasing: as the confidence $\hat{p}$ increases toward 1.0, the entropy H(𝐩) decreases toward 0, and conversely. This inverse relationship defines a curved lower boundary in the $\left(\hat{p},H\right)$ plane that corresponds to the degenerate case where all remaining probability mass $1-\hat{p}$ is distributed uniformly across the remaining C−1=9 classes. For this degenerate case, the entropy as a function of confidence is given by:


$$\begin{array}{c} H_{\text{boundary}}(\hat{p})=-\hat{p}\ln(\hat{p})-(1-\hat{p})\ln\;\left(\frac{1-\hat{p}}{C-1}\right) \end{array}$$ (52)

All empirically observed $\left(\hat{p},H\right)$ pairs must lie on or above this boundary curve, as this curve represents the minimum entropy achievable for a given confidence value.

#### Confidence distribution stratified by correctness.

The average prediction confidence computed separately over correct and incorrect predictions is:


$$\begin{array}{c} {\bar{p}}_{\text{correct}}=\frac{1}{\left|𝒞\right|}\sum_{i\in 𝒞}{\hat{p}}^{\left(i\right)}=0.9612 \end{array}$$ (53)

$$\begin{array}{c} {\bar{p}}_{\text{incorrect}}=\frac{1}{\left|𝒲\right|}\sum_{i\in 𝒲}{\hat{p}}^{\left(i\right)}=0.7302 \end{array}$$ (54)

where $𝒞=\{i:{\hat{y}}^{\left(i\right)}=y^{\left(i\right)}\}$ denotes the set of correctly classified samples and $𝒲=\{i:{\hat{y}}^{\left(i\right)}\neq y^{\left(i\right)}\}$ denotes the set of incorrectly classified samples. The absolute confidence gap between correct and incorrect predictions is:


$$\begin{array}{c} \Delta \bar{p}={\bar{p}}_{\text{correct}}-{\bar{p}}_{\text{incorrect}}=0.9612-0.7302=0.2310 \end{array}$$ (55)

**Fig 15:**
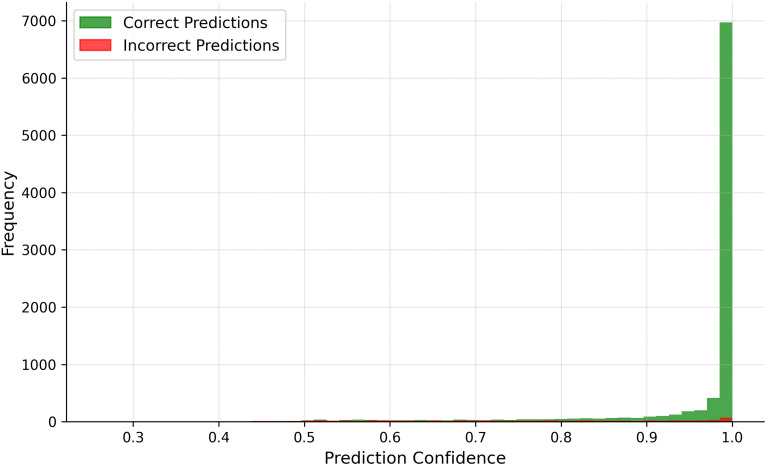
Prediction confidence distribution stratified by correctness. Overlapping histograms of prediction confidence for correct (green) and incorrect (red) samples.

This gap of approximately 0.23 indicates that the model is substantially more confident when its predictions are correct than when they are incorrect, which is a desirable property consistent with a well-calibrated classifier. The confidence distribution histogram presented in Fig 15 reveals the full distributional picture underlying these summary statistics. The distribution of correct prediction confidences (green bars) is strongly right-skewed, with the overwhelming majority of correctly classified samples concentrated in the highest confidence bin approaching $\hat{p}\approx 1.0$, with a frequency exceeding 7000 samples. The distribution of incorrect prediction confidences (red bars) is substantially broader and more diffuse, with meaningful mass distributed across the range $\hat{p}\in [0.25,0.5]$, reflecting the fact that incorrect predictions are associated with considerably more distributional uncertainty. The near-zero frequency of incorrect predictions at very high confidence values ($\hat{p}>0.7$) suggests that overconfident misclassifications are rare, which is an important property indicating that the model does not systematically assign high confidence to incorrect predictions.

#### Per-class average confidence.

**Fig 16:**
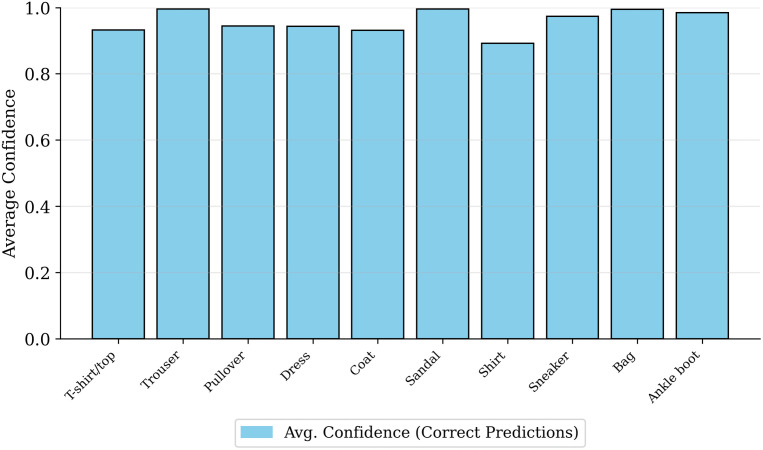
Average prediction confidence ${\bar{p}}_{c}$ per class over correctly classified samples. Bar chart of mean softmax confidence per class, showing consistently high confidence (>0.90) across all ten Fashion MNIST classes, with Shirt yielding the lowest average confidence.

To examine whether confidence varies systematically across the ten Fashion MNIST classes, the average confidence is computed separately for each class *c* over the subset of correctly classified samples belonging to that class:


$$\begin{array}{c} {\bar{p}}_{c}=\frac{1}{\left|{𝒞}_{c}\right|}\sum_{i\in {𝒞}_{c}}{\hat{p}}^{\left(i\right)},\;c=1,\ldots ,C \end{array}$$ (56)

where ${𝒞}_{c}=\{i\in 𝒞:y^{\left(i\right)}=c\}$ denotes the set of correctly classified samples belonging to class *c*. The per-class confidence bar chart presented in Fig 16 reveals substantial variation across classes. Visually distinctive and structurally unambiguous classes, specifically Trouser and Sandal, achieve the highest average confidence values of approximately ${\bar{p}}_{c}\approx 1.00$, consistent with their low inter-class visual similarity and the expectation that the model can identify these classes with high certainty. In contrast, Shirt achieves the lowest average confidence of approximately ${\bar{p}}_{\text{Shirt}}\approx 0.89$, reflecting the high degree of visual overlap between Shirt and neighbouring upper-body garment classes including T-shirt/top, Pullover, and Coat. T-shirt/top and Coat also exhibit notably lower average confidence values of approximately $\bar{p}\approx 0.93$, consistent with their frequent confusion with Shirt. The per-class confidence values may be interpreted as a class-level indicator of discriminability: classes with ${\bar{p}}_{c}\approx 1.0$ are well-separated from all other classes in the model’s learned feature space, while classes with lower ${\bar{p}}_{c}$ occupy more ambiguous regions of the feature space where the decision boundaries are less well-defined.

#### Top confusion pairs.

**Fig 17:**
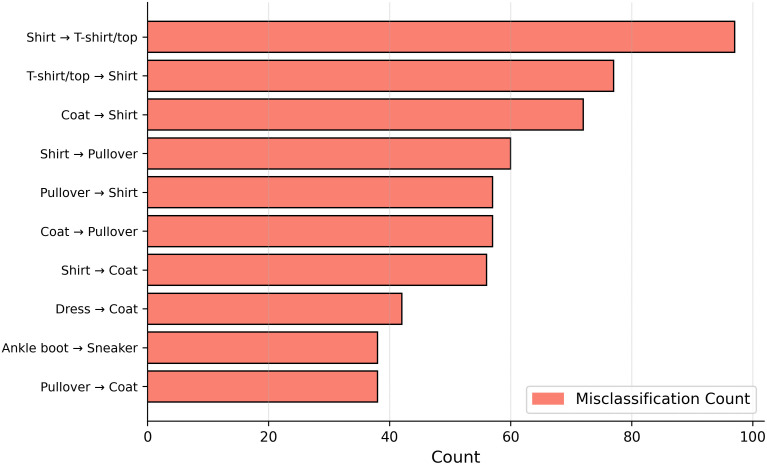
Top ten most frequent misclassification pairs. Each bar represents the count of samples misclassified from the true class (left) to the predicted class (right).

To identify the specific class pairs responsible for the majority of prediction errors, the frequency of each ordered misclassification pair $\left(y,\hat{y}\right)$ with $y\neq \hat{y}$ is counted across all incorrect predictions. The top ten most frequent confusion pairs are presented in Fig 17. The dominant confusion pair is Shirt → T-shirt/top with approximately 97 instances, followed by T-shirt/top → Shirt with approximately 77 instances, and Coat → Shirt with approximately 73 instances. Shirt → Pullover and Pullover → Shirt each contribute approximately 60 and 57 instances respectively, while Coat → Pullover and Shirt → Coat each contribute approximately 57 and 56 instances. The remaining top ten pairs include Dress → Coat with approximately 42 instances, Ankle boot → Sneaker with approximately 39 instances, and Pullover → Coat with approximately 39 instances. Collectively, the top ten confusion pairs are almost entirely concentrated within the upper-body garment cluster comprising Shirt, T-shirt/top, Pullover, and Coat, with the single exception of Ankle boot → Sneaker. This concentration is consistent with the per-class confidence analysis: the classes involved in the most frequent confusion pairs are precisely those exhibiting the lowest average confidence values in Fig 16, namely Shirt ($\bar{p}\approx 0.89$), T-shirt/top ($\bar{p}\approx 0.93$), and Coat ($\bar{p}\approx 0.93$).

A notable structural feature of the confusion pattern is its bidirectionality within the upper-body garment cluster: both Shirt → T-shirt/top and T-shirt/top → Shirt appear in the top ten, as do Shirt → Pullover and Pullover → Shirt, and Shirt → Coat and Coat → Shirt. This bidirectionality indicates that the confusion arises from genuine inter-class visual ambiguity in the shared feature space rather than from a systematic directional bias in the model’s decision boundaries. The confusion between Ankle boot and Sneaker is similarly interpretable as arising from shared silhouette and structural features between the two footwear classes.

#### Confidence versus entropy scatter plot.

**Fig 18:**
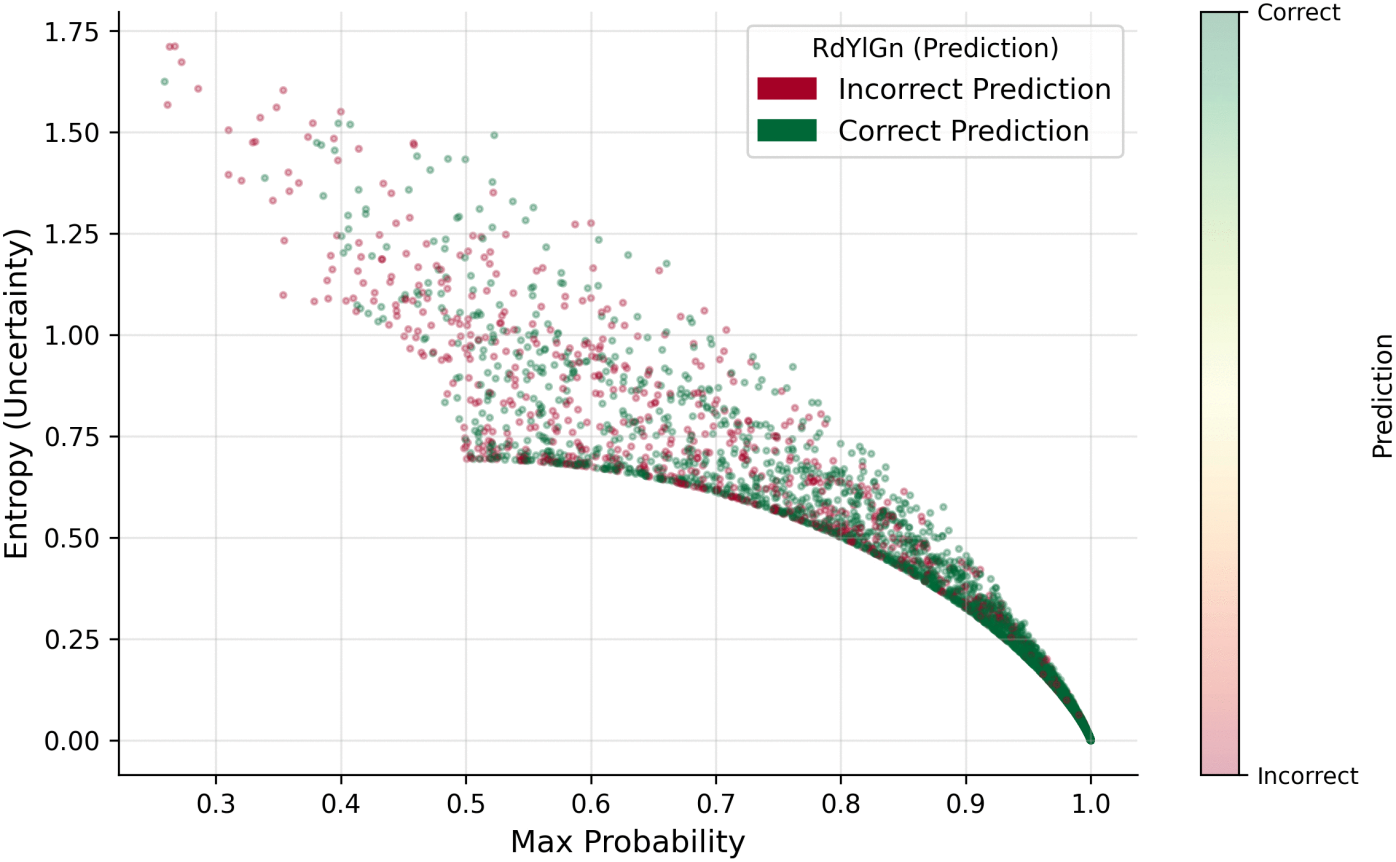
Max probability versus entropy, colored by correctness. Each point represents one test sample colored by prediction correctness (green: correct, red: incorrect), showing max softmax probability on the x-axis against prediction entropy on the y-axis.

The scatter plot of prediction confidence $\hat{p}$ against prediction entropy H(𝐩) presented in Fig 18 provides a joint visualization of the two complementary uncertainty measures across all test samples, with each point coloured according to prediction correctness using the *RdYlGn* colormap, where green denotes correctly classified samples and pink/red denotes incorrectly classified samples. The curved lower boundary of the scatter cloud is determined by Eq 52 and represents the minimum entropy achievable for a given confidence value. As $\hat{p}\to 1.0$, this boundary approaches zero, and as $\hat{p}\to 1/C=0.1$, this boundary approaches the maximum entropy of ln(10)≈2.303 nats. The dense cluster of green points along the lower boundary at high confidence values ($\hat{p}>0.9$) reflects the strongly right-skewed distribution of correct prediction confidences observed in Fig 15, where the majority of correctly classified samples achieve near-unity confidence with correspondingly near-zero entropy.

The scatter plot reveals a clear spatial separation between correct and incorrect predictions in the $\left(\hat{p},H\right)$ plane. Incorrect predictions are disproportionately concentrated in the high-entropy, low-confidence region corresponding to $\hat{p}≲0.6$ and $\begin{array}{c} H≳1.0 \end{array}$ nats, while correct predictions dominate the low-entropy, high-confidence region corresponding to $\begin{array}{c} \hat{p}≳0.9 \end{array}$ and H≲0.5 nats. This separation is consistent with the summary statistics reported in Eq 53 and q 54. However, a non-trivial overlap zone is evident in the intermediate confidence range $\hat{p}\in [0.5,0.9]$, where both correct and incorrect predictions coexist with comparable density. This overlap zone represents the region of the probability simplex where the model’s discriminative ability is least reliable, and may indicate samples for which the input lies near a decision boundary in the learned feature space. The existence of this overlap zone suggests that confidence thresholding alone, for example by abstaining from prediction when $\hat{p}<\tau$ for some threshold τ, would not achieve perfect separation between correct and incorrect predictions, and that more sophisticated uncertainty quantification approaches such as temperature scaling, Bayesian inference, or Monte Carlo Dropout may be required to achieve better-calibrated uncertainty estimates in this intermediate confidence regime.

### Quantitative comparison of XAI methods

The qualitative analyses of Integrated Gradients, LIME, and SHAP attribution maps has been presented in the preceding sections which provided sample-level visual evidence of the explanatory behaviour of each method. This section presents a quantitative comparison of the three XAI methods across *N* = 200 randomly selected test samples, measuring pairwise agreement via Spearman rank correlation and top-*k*% pixel overlap, and measuring individual faithfulness via the deletion probability drop metric.

#### Implementation.

For each of the *N* = 200 test samples, the predicted class label is obtained by passing the image through the full TinyCNN-LSA model and taking the argmax of the logit vector, as defined in Eq 47. Three attribution maps are then computed for the predicted class using Integrated Gradients, LIME, and SHAP respectively, and two quantitative metrics, Pairwise Agreement and Faithfulness, are computed from these maps.

#### Integrated gradients.

The IG attribution map is computed with a zero-valued baseline ${𝐱}^{\prime }=0$ and n_steps=24 approximation steps. The absolute value of the attribution is taken to produce a non-negative importance map of shape 28×28.

#### LIME.

The LIME attribution map is computed by perturbing the input image 250 times with randomly masked superpixel regions and observing how the model’s predicted class probability changes across these perturbations. The image is first replicated across three channels to satisfy LIME’s RGB input requirement, and then divided into small contiguous regions called superpixels using the quickshift segmentation algorithm with parameters kernel_size=2, max_dist=5, and *ratio* = 0.5. LIME fits a local linear model to the relationship between which superpixels are visible and the model’s output probability, and the resulting coefficients indicate how much each superpixel region supports or opposes the predicted class. A positive coefficient means that region pushed the model toward the predicted class, while a negative coefficient means it pushed the model away. These per-superpixel importance scores are then painted back onto the image so that every pixel within a superpixel inherits its region’s score, producing a dense 28×28 importance map. The absolute value is finally taken so that both supporting and opposing regions are treated as important, regardless of the direction of their influence.

#### SHAP.

The SHAP attribution map is computed using a gradient-based approach that compares how the model responds to the input image versus how it responds to a set of reference images. Specifically, 50 randomly selected training images are used as a background reference dataset, representing a baseline of typical inputs the model has seen during training. For each test image, SHAP measures how much each pixel causes the model’s predicted class probability to deviate from what would be expected on average across these 50 background images. The resulting importance scores are extracted for the predicted class only, and the absolute value is taken so that pixels that either strongly increase or strongly decrease the predicted probability are both treated as important, producing a non-negative 28×28 attribution map.

#### Pairwise agreement metrics.

For each pair of attribution maps $\left(f_{A},f_{B})\in \{(\text{IG},\text{LIME}),\;(\text{IG},\text{SHAP}),\;(\text{LIME},\text{SHAP})\right\}$, two agreement metrics are computed. The Spearman rank correlation is computed between the flattened attribution vectors $𝐚=f_{A}(𝐱)\in {\mathbb{R}}^{784}$ and $𝐛=f_{B}(𝐱)\in {\mathbb{R}}^{784}$ as:


$$\begin{array}{c} \rho_{AB}=\text{Spearman}\;\left(𝐚,\;𝐛\right)=1-\frac{6\sum_{i=1}^{784}d_{i}^{2}}{784\;(784^{2}-1)} \end{array}$$ (57)

where $d_{i}=\text{rank}(a_{i})-\text{rank}(b_{i})$ is the difference in ranks of the *i*-th pixel attribution between the two maps, implemented via scipy.stats.spearmanr. The Spearman correlation is preferred over the Pearson correlation here because it is invariant to monotonic rescaling of the attribution values and is therefore robust to the different absolute scales produced by the three XAI methods. A value of $\rho_{AB}=1$ indicates perfect rank agreement, $\rho_{AB}=0$ indicates no monotonic relationship, and $\rho_{AB}=-1$ indicates perfect rank disagreement.

The top-*k*% pixel overlap is computed as the Jaccard-like intersection ratio of the sets of the top-*k*% most important pixels identified by each method:


$$\begin{array}{c} {\text{Overlap}}_{AB}=\frac{\left|{𝒯}_{A}^{\left(k\right)}\cap {𝒯}_{B}^{\left(k\right)}\right|}{k} \end{array}$$ (58)

where k=⌊0.10×784⌋=78 pixels corresponds to the top 10% of the 784 total pixels, and ${𝒯}_{A}^{\left(k\right)}$ and ${𝒯}_{B}^{\left(k\right)}$ denote the sets of indices of the *k* highest-attribution pixels under methods *A* and *B* respectively, obtained by ranking all pixel attribution values from lowest to highest and selecting the indices of the *k* largest values. A value of ${\text{Overlap}}_{AB}=1.0$ indicates that the two methods identify exactly the same top-10% pixels, while ${\text{Overlap}}_{AB}=0$ indicates no overlap. Under a uniform random baseline, the expected overlap between two independently drawn sets of *k* pixels from a pool of *P* = 784 is:


$$\begin{array}{c} 𝔼[{\text{Overlap}}_{\text{random}}]=\frac{k}{P}=\frac{78}{784}\approx 0.099 \end{array}$$ (59)

providing a natural reference point against which the observed overlap values may be compared.

#### Faithfulness via deletion.

The faithfulness of each attribution map is evaluated using the deletion metric, which measures the drop in the model’s predicted class probability after masking the top-*k*% most important pixels identified by the attribution method with a zero baseline value:


$$\begin{array}{c} \Delta p^{\left(m\right)}=p_{\hat{y}}(𝐱)-p_{\hat{y}}\;\left(𝐱⊙(1-{𝐌}^{\left(m\right)})\right) \end{array}$$ (60)

where $p_{\hat{y}}(𝐱)$ is the original predicted class probability, ${𝐌}^{\left(m\right)}\in \{0,1{\}}^{784}$ is a binary mask with ones at the top-*k* = 78 pixel positions identified by method m∈{IG,LIME,SHAP}, and ⊙ denotes elementwise multiplication. The masked pixels are set to the baseline value of 0.0 (corresponding to a black pixel in the normalised image space). A higher value of $\Delta p^{\left(m\right)}$ indicates that the pixels identified as most important by method *m* are genuinely more influential for the model’s prediction, reflecting higher faithfulness of the attribution map to the model’s actual decision process. All metrics are computed per sample and aggregated across the *N* = 200 samples to produce mean and standard deviation estimates.

#### Pairwise agreement results.

**Fig 19:**
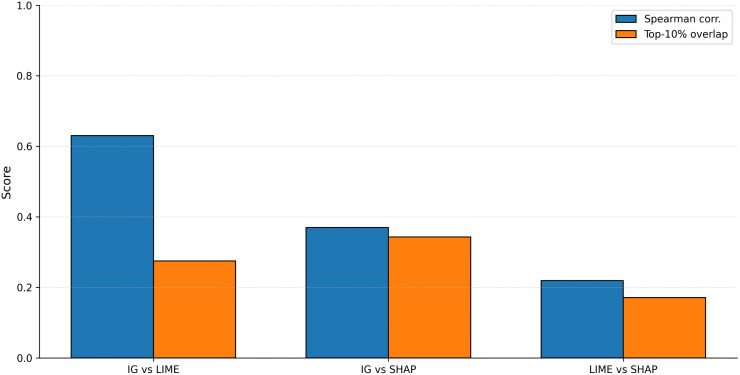
Pairwise XAI agreement over 200 test samples: Spearman correlation and top-10% pixel overlap between IG, LIME, and SHAP. Each group shows Spearman correlation (blue) and top-10% pixel overlap (orange) between each pair of XAI methods across 200 test samples.

The pairwise agreement results are presented in Table 4 and Fig 19. The highest pairwise agreement is observed between IG and LIME, with a Spearman correlation of $\rho_{\text{IG,LIME}}=0.630\pm 0.160$ and a top-10% overlap of ${\text{Overlap}}_{\text{IG,LIME}}=0.275\pm 0.135$. The Spearman correlation of 0.630 indicates a moderate positive rank agreement between the pixel-level importance rankings produced by the two methods, suggesting that IG and LIME tend to assign relatively consistent importance orderings to pixels across the 28×28 image. The top-10% overlap of 0.275 indicates that approximately 27.5% of the top-78 pixels identified by IG are also identified as top-78 pixels by LIME, which is approximately 2.78 times the random baseline expectation of 0.099 from Eq 59:


$$\begin{array}{c} \frac{{\text{Overlap}}_{\text{IG,LIME}}}{𝔼[{\text{Overlap}}_{\text{random}}]}=\frac{0.275}{0.099}\approx 2.78 \end{array}$$ (61)

**Table 4:** Pairwise XAI agreement over *N* = 200 test samples.

Pair	Spearman correlation (mean ± std)	Top-10% overlap (mean ± std)
IG vs LIME	0.630±0.160	0.275±0.135
IG vs SHAP	0.370±0.163	0.343±0.128
LIME vs SHAP	0.219±0.152	0.171±0.086

The second highest agreement is observed between IG and SHAP, with $\rho_{\text{IG,SHAP}}=0.370\pm 0.163$ and ${\text{Overlap}}_{\text{IG,SHAP}}=0.343\pm 0.128$. Notably, while the Spearman correlation between IG and SHAP is lower than that between IG and LIME, the top-10% overlap between IG and SHAP is higher, at 0.343 compared to 0.275. This apparent discrepancy suggests that IG and SHAP agree more strongly on which pixels belong to the top-10% most important set, but disagree more on the precise ranking of those pixels within that set. The top-10% overlap of 0.343 corresponds to approximately 3.46 times the baseline:


$$\begin{array}{c} \frac{{\text{Overlap}}_{\text{IG,SHAP}}}{𝔼[{\text{Overlap}}_{\text{random}}]}=\frac{0.343}{0.099}\approx 3.46 \end{array}$$ (62)

The lowest pairwise agreement is observed between LIME and SHAP, with $\rho_{\text{LIME,SHAP}}=0.219\pm 0.152$ and ${\text{Overlap}}_{\text{LIME,SHAP}}=0.171\pm 0.086$. The Spearman correlation of 0.219 reflects only weak rank agreement between the two methods, and the top-10% overlap of 0.171 is approximately 1.73 times the random baseline:


$$\begin{array}{c} \frac{{\text{Overlap}}_{\text{LIME,SHAP}}}{𝔼[{\text{Overlap}}_{\text{random}}]}=\frac{0.171}{0.099}\approx 1.73 \end{array}$$ (63)

indicating only modest agreement beyond chance. The relatively low agreement between LIME and SHAP is consistent with the fundamental methodological differences between the two approaches: LIME produces superpixel-level attributions based on local linear approximations using random occlusion perturbations, while SHAP produces pixel-level attributions based on gradient information with respect to a background reference dataset. These differences in attribution granularity and perturbation strategy contribute to the lower observed agreement between LIME and SHAP compared to the gradient-based IG and SHAP pair, which share a common gradient-based computational foundation.

The standard deviations reported for all three pairs are substantial relative to the means, ranging from 0.152 to 0.163 for the Spearman correlation and from 0.086 to 0.135 for the top-10% overlap. This variability indicates that the degree of agreement between methods is not uniform across samples, and may vary substantially depending on the input image, the predicted class, and the complexity of the local decision boundary in the feature space.

#### Faithfulness results.

**Fig 20:**
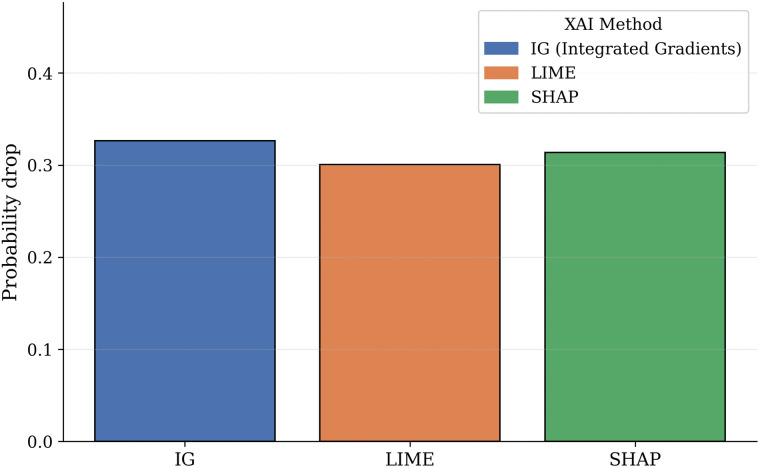
Mean probability drop after masking the top-10% highest-attributed pixels for IG, LIME, and SHAP over 200 test samples. Each bar shows the mean prediction probability drop per XAI method when the top-10% highest-attributed pixels are masked from the input.

Faithfulness scores are reported in Table 5 and Fig 20. The faithfulness results are measured as the mean probability drop $\Delta p^{\left(m\right)}$ after masking the top-10% attributed pixels. SHAP achieves the highest mean faithfulness of ${\overset{―}{\Delta p}}^{\text{SHAP}}=0.352\pm 0.393$, indicating that masking the top-78 pixels identified by SHAP causes an average reduction of approximately 0.352 in the model’s predicted class probability. Integrated Gradients achieves the second highest faithfulness of ${\overset{―}{\Delta p}}^{\text{IG}}=0.327\pm 0.399$, and LIME achieves the lowest faithfulness of ${\overset{―}{\Delta p}}^{\text{LIME}}=0.301\pm 0.421$. The relative faithfulness ordering may be summarised as:


$$\begin{array}{c} {\overset{―}{\Delta p}}^{\text{SHAP}}>{\overset{―}{\Delta p}}^{\text{IG}}>{\overset{―}{\Delta p}}^{\text{LIME}}\;⟺\;0.352>0.327>0.301 \end{array}$$ (64)

**Table 5:** Faithfulness scores after masking the top-10% attributed pixels over 200 test samples.

Method	Probability drop (mean ± std)
IG	0.327±0.399
LIME	0.301±0.421
SHAP	0.352±0.393

The highest faithfulness of SHAP is consistent with its gradient-based attribution mechanism, which computes pixel importance as the expected deviation from a set of 50 background reference images. By anchoring attributions relative to a distribution of typical training inputs rather than a single fixed baseline, SHAP may more reliably identify pixels whose removal causes the largest shift in predicted probability. Integrated Gradients achieves comparable faithfulness, which is also expected given its gradient-based nature: IG integrates gradients along a path from a zero baseline to the input and is therefore directly sensitive to pixels that most strongly influence the model’s output. The lower faithfulness of LIME relative to both gradient-based methods reflects the superpixel-level granularity of LIME attributions, which assigns uniform importance to all pixels within a superpixel region and may therefore include pixels that are not individually important for the model’s prediction within the top-10% set.

To contextualise the observed faithfulness values, it is useful to note that a completely random attribution map would be expected to produce a probability drop proportional to the fraction of pixels masked, scaled by the average pixel importance. For a model that distributes its sensitivity uniformly across all 784 pixels, masking 10% of pixels would be expected to produce a probability drop of approximately 10% of the original predicted probability. The observed mean probability drops of 0.352, 0.327, and 0.301 for SHAP, IG, and LIME respectively are substantially larger than this naive expectation, confirming that all three methods identify genuinely important pixels rather than selecting pixels at random, and that the top-10% pixels identified by each method carry disproportionate importance for the model’s predictions. The experiment used a fixed random seed of 42.

## Results

The proposed TinyCNN + Linear Self-Attention (LSA) hybrid architecture was evaluated on the Fashion-MNIST dataset to analyze its classification performance and computational efficiency. The model has fewer than 0.5 million parameters, significantly lower than models like ResNet-18 (11.7M), ResNet-50 (25.6M), VGG16 (138M), MobileNetV1 (4.2M), and MobileNetV2 (3.4M), yet achieved 91.47% test accuracy. The TinyCNN-LSA model achieved a high training accuracy of 94.41%, while maintaining appropriate generalization on the validation (92.12%) and test sets. The relatively small gap between training and test accuracies indicates effective regularization and suggests that the self-attention module enhanced the network’s capacity to extract semantically meaningful global features. To improve generalization and prevent overfitting, a dropout layer with a 0.3 rate is used between the classifier’s fully connected layers. The training still converged efficiently at epoch 26, which means dropout did not hinder convergence. The training successfully finished in 2510.23 seconds albeit without a GPU. Epoch’s average duration was 96.55 seconds. Early stopping was triggered at epoch number 26, this indicates efficient convergence behavior of the model. The model also showed a fast inference performance, with a latency of mere 0.90 milliseconds per test sample. Table 6 presents a comparative evaluation of the TinyCNN-LSA model against a few models inlcuding LSTM [29], LSTM (Shen) [30], VGG11 [31], VGG19 and ResNet50 [32], SVM + HOG [33], MobileNetV1 [25], MobileNetV2 [25], and Fusion Model (VGG16 + ResNet50 + MobileNetV2) [26]. A comparison of precision, recall, and F1-score across LSTM-based models on the Fashion MNIST dataset is presented in Table 7. Zhang’s LSTM model reported a precision of 89.74%. Shen’s LSTM model reported an overall precision, recall, and F1-score of 88.87%, 88.56%, and 88.56%, respectively. The proposed model outperforms both prior works across all reported metrics, achieving a precision, recall, and F1-score of 91.00% each, demonstrating its better classification capability on the Fashion MNIST dataset. Table 8 presents a comparison of per-class correct predictions across models evaluated on the Fashion MNIST test set, each containing 1,000 samples per class.

**Table 6:** TinyCNN-LSA vs. Existing Models on Fashion-MNIST.

Model	Test Acc. (%)	Val. Acc. (%)	Train Acc. (%)	Val. Loss	Train Loss	Latency (ms)
LSTM	89.94	–	–	–	–	–
VGG11	91.50	–	–	–	–	–
VGG19	91.92	–	–	–	–	5.439
ResNet50	90.25	–	–	–	–	3.522
LSTM (Shen)	88.26	–	–	–	–	–
SVM + HOG	86.53	–	–	–	–	–
MobileNetV1	91.12	–	–	–	–	–
MobileNetV2	92.91	–	–	–	–	–
Fusion Model	–	89.27	97.50	–	–	–
**TinyCNN-LSA**	**91.47**	**92.12**	**94.41**	**0.2373**	**0.1483**	**0.90**

**Table 7:** Performance comparison on Precision, Recall and F1-Score.

Model	Precision	Recall	F1-Score
LSTM	89.74%	—	—
LSTM (Shen)	88.87%	88.56%	88.56%
**TinyCNN-LSA**	**91.00%**	**91.00%**	**91.00%**

**Table 8:** Number of Correctly Classified Samples per Class Across Models on the Fashion-MNIST Test Set.

Class	TinyCNN-LSA	LSTM	LSTM (Shen)	VGG19	ResNet50	HOG + SVM	Fusion Model
T-Shirt/Top	**873**	802	813	856	881	835	774
Trouser	**989**	986	960	990	972	963	941
Pullover	**883**	838	793	916	849	765	730
Dress	**902**	899	889	949	894	871	885
Coat	**853**	859	793	887	882	796	786
Sandal	**977**	974	959	990	984	950	913
Shirt	**752**	741	731	748	682	612	460
Sneaker	**975**	956	943	969	973	952	866
Bag	**986**	977	974	989	987	965	959
Ankle Boot	**957**	962	971	972	964	944	929
**Total Correct**	**9,147**	8,994	8,826	9,266	9,068	8,653	8,243

### Training stability and convergence

**Fig 21:**
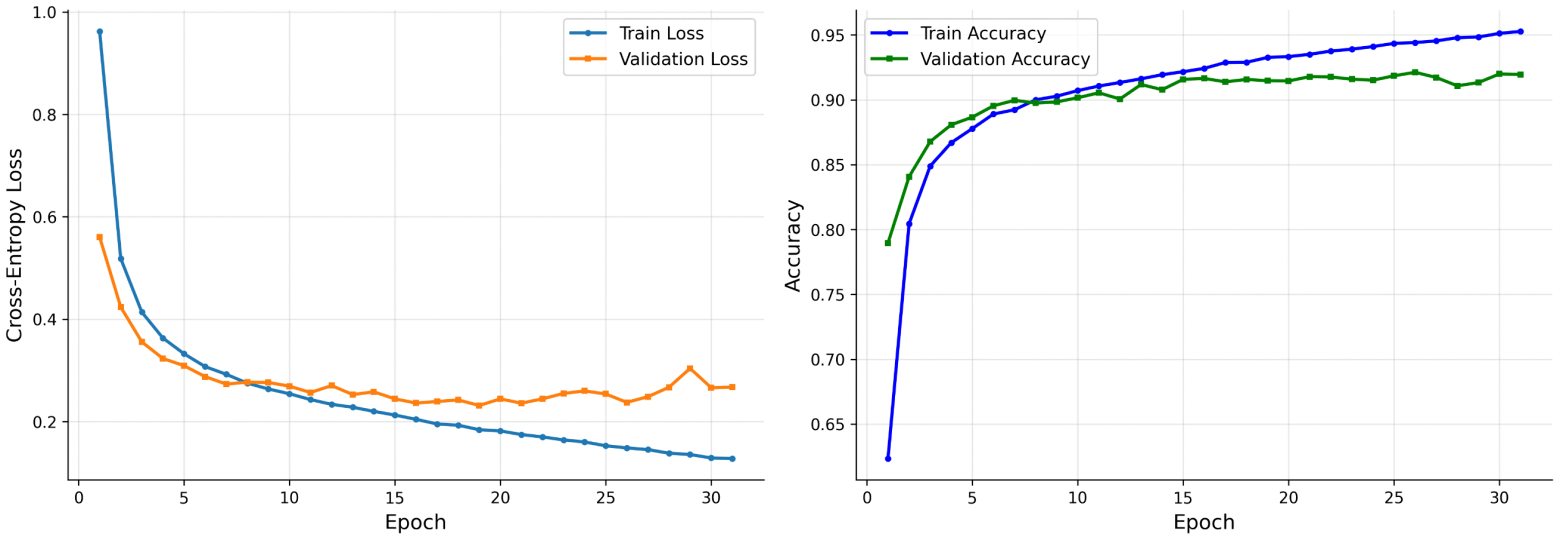
Accuracy and Loss curve of TinyCNN-LSA Model. The left panel shows training and validation cross-entropy loss across epochs, while the right panel shows training and validation accuracy across epochs.

There is a steady improvement in accuracy across epochs, and a decrease in the training and validation losses over epochs, which indicates that the model’s learning is smooth and consistent. Fig 21 represents the training and validation accuracy and loss graphs.

### Classification report

In Table 9, the detailed classification metrics for each fashion category are displayed, where precision is the ratio of correctly predicted positive observations to the total predicted positives (i.e., it measures how accurate the model is), recall is the ratio of correctly predicted positive observations to all actual positives (i.e., it determines how well a model can find an object) and F1-score is a weighted average of precision and recall. The model performs remarkably well on some categories. The metrics for both Trouser and Bag reach nearly perfect classification with scores of 0.99 in all cases. Whereas Sandal (0.98), Ankle boot (0.97), and Sneaker (0.96) are just as good or even better. High performing classes can always be identified by one common characteristic, they have unique shape and attributes that sets them apart from all other classes. Footwears and bags have their own specific structure so that they are easily recognizable to the model. On the other hand, Shirt is the most difficult category with the lowest measures (precision: 0.76, recall: 0.75, F1-score: 0.76). This problem arises from the visual similarity between shirts and other upper-body clothing such as T-shirts, pullovers or coats. Similar reasons account for the relatively lower performance (0.85) on the Coat category as well. For wavy patterns T-shirt/top and Pullover both get average scores of 0.87 whereas Dress gets a good score 0.91. In summary, the overall macro average of 0.91 across all metrics represents a better model performance that model has learned meaningful features.

**Table 9:** Classification Report.

Class	Precision	Recall	F1-Score	Support
T-shirt/top	0.87	0.87	0.87	1000
Trouser	0.99	0.99	0.99	1000
Pullover	0.86	0.88	0.87	1000
Dress	0.93	0.90	0.91	1000
Coat	0.85	0.85	0.85	1000
Sandal	0.99	0.98	0.98	1000
Shirt	0.76	0.75	0.76	1000
Sneaker	0.95	0.97	0.96	1000
Bag	0.99	0.99	0.99	1000
Ankle boot	0.97	0.96	0.97	1000
**Macro Avg**	0.91	0.91	0.91	10000
**Weighted Avg**	0.91	0.91	0.91	10000

### Confusion matrix

**Fig 22:**
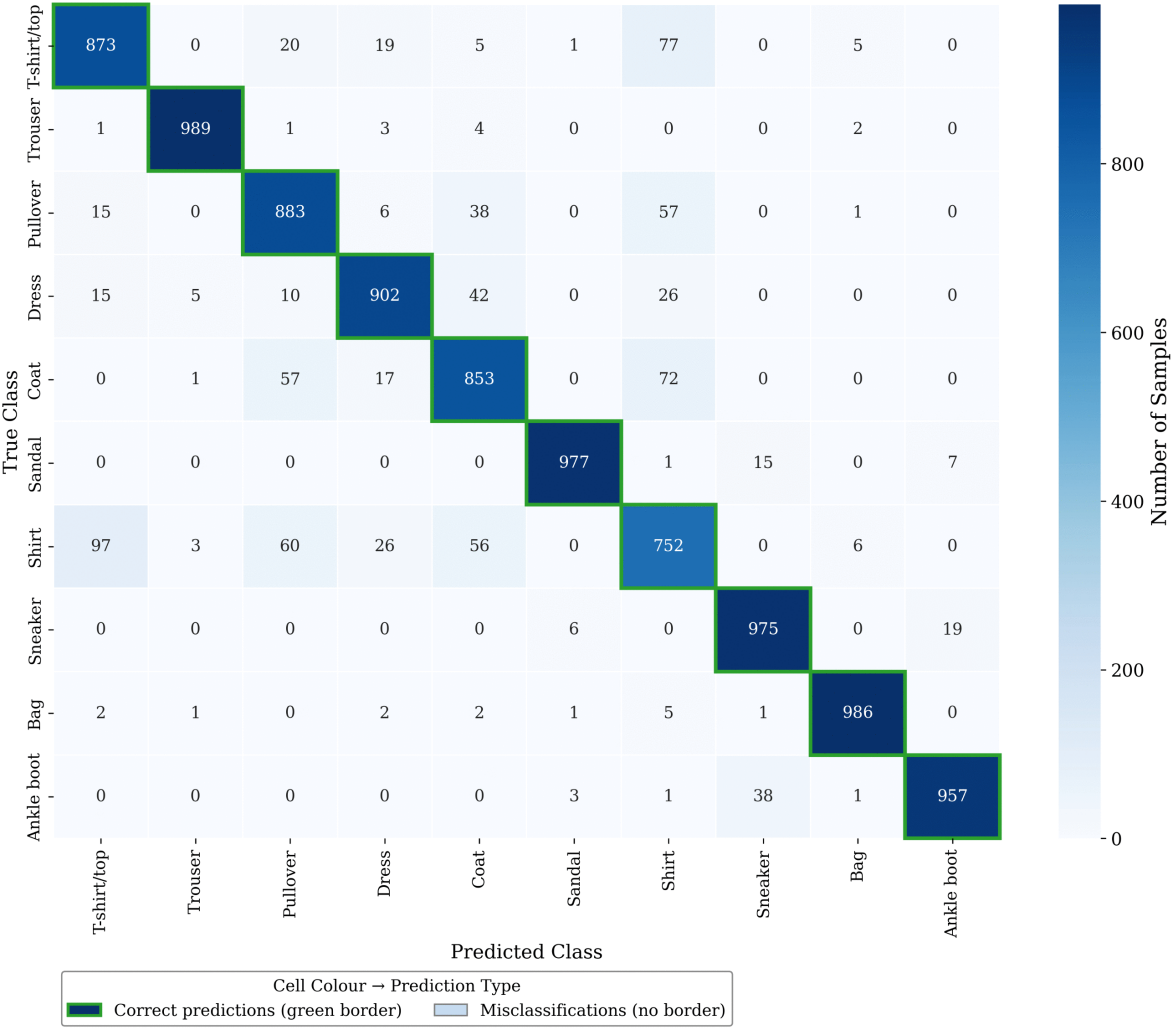
Confusion matrix. The x-axis shows the predicted class and the y-axis shows the true class; diagonal cells represent correct predictions, while non-diagonal cells represent misclassifications (cell values are sample counts).

The confusion matrix in Fig 22 shows the classification performance of the proposed model on the test set for all ten fashion categories. The diagonal entries correspond to the true positives and the off-diagonal elements represent false negatives and false positives. These results indicate that the classifier does really well for some classes. With 989 out of 1000 correct predictions, Trouser is virtually indistinguishable from other categories. The same applies for Sandal (977), Sneaker(975), Bag(986)and Ankle boot (957) which are performing pretty well due to unique object features visually that can be clearly distinguished from other clothing type. Yet the matrix shows some confusion between visually similar upper-body items. T-shirt/top demonstrates misclassification with Shirt (77 samples), probably due to their visual similarity. Similarly, Shirt is often confused with T-shirt/top (97 cases), Pullover (60) and Coat (56). Pullover (57) and Shirt (72) in the Coat category also shows confusion due to the similar visual appearance of layered clothing. In footwear categories, some cross-confusion exists as well which is not surprising, such that Sneaker is being classified as Ankle boot and vice versa in some cases (19 times vs. 38). These problems with visually similar items may explain some of the low off-diagonal values, but the high diagonal values suggest that the overall learned features are discriminatory for most categories.

## Discussion

The results of this study indicate that a compact hybrid architecture combining convolutional feature extraction with linear self-attention offers a reasonable approach to image classification in settings where computational resources are a consideration. This section discusses the broader implications of this research in terms of conceptual design, practical deployment, and interpretability.

In terms of conceptual design, it is worth situating this research within the broader landscape of existing hybrid architectures. Models that combine MobileNet or EfficientNet backbones with attention mechanisms generally incorporate channel-wise or squeeze-and-excitation style attention, which reweights feature channels rather than modeling spatial relationships across the full feature map [34]. The approach taken in this study differs in that the linear self-attention module operates across all spatial positions of the feature map, allowing the model to capture broader spatial context. Architectures such as ConViT and MobileViT incorporate transformer components with positional encodings and patch tokenization pipelines, which introduce additional design complexity [35]. This study does not rely on patch tokenization or positional encodings, instead allowing the convolutional layers to encode spatial structure implicitly, and applying attention directly to the resulting feature representations. This keeps the overall architecture straightforward while retaining the capacity to model spatial relationships across the feature map.

A further distinction is the use of linear self-attention over standard softmax attention. The linear self-attention formulation used in this study approximates the attention operation in a way that scales linearly with the number of spatial tokens, as opposed to the quadratic scaling of standard attention [36]. This is a property that is appropriate for settings where efficiency is a priority and represents a meaningful difference from the attention mechanisms used in many existing CNN-Transformer hybrid models.

In terms of practical implications, the linear scaling behavior of the attention module means that computational cost remains proportional to input size, which is suitable for tasks where spatial resolution varies. The absence of patch tokenization and positional encodings also means the architecture involves fewer preprocessing assumptions, making it more straightforward to apply across different types of visual data. The combination of a convolutional backbone with a single linear self-attention block results in a model that is modest in parameter count and appropriate for settings where resource availability is a practical consideration.

## Conclusion

This research presents a lightweight hybrid model that combines Linear Self-Attention with a compact TinyCNN backbone for fashion image classification on the Fashion-MNIST dataset. The proposed TinyCNN-LSA architecture contains fewer than half a million parameters and is designed to be trainable on a CPU without the need for dedicated GPU hardware, achieving a classification accuracy of 91.47%. These results demonstrate that the architecture offers a practical balance between classification performance and computational efficiency, making it a viable option for deployment in low-resource environments. TinyCNN-LSA is intentionally designed to be compact, sacrificing some representational capacity in exchange for significantly reduced computational cost and memory footprint. Despite this trade-off, the integration of Linear Self-Attention allows the model to capture global contextual dependencies within the image that traditional CNNs inherently lack, partially compensating for the reduced depth and helping the model achieve competitive classification accuracy on Fashion-MNIST. The XAI techniques applied in this study, including Self-Attention visualization, Multi-Head Attention, Attention Flow, Attention Rollout, Fixed Query Position Attention Maps, Integrated Gradients, LIME, and SHAP, further enhance the model’s transparency by providing visual interpretability of its predictions, which is particularly valuable in real-world applications where model decisions must be understood and trusted. Future work would explore evaluating the model’s scalability and generalization performance on higher-resolution images and more complex datasets.
